# Multipronged regulation of autophagy and apoptosis: emerging role of TRIM proteins

**DOI:** 10.1186/s11658-023-00528-8

**Published:** 2024-01-16

**Authors:** Nuzhat Ahsan, Mohd Shariq, Avadhesha Surolia, Reshmi Raj, Mohammad Firoz Khan, Pramod Kumar

**Affiliations:** 1 Quantlase Lab LLC, Unit 1-8, Masdar City, Abu Dhabi, UAE; 2 Molecular Biophysics Unit, Indian Institute of Science, Bangalore, 460012 India

**Keywords:** TRIM proteins, E3-Ub ligase, Apoptosis, Autophagy, Ubiquitination, Autophagosome, BECN1, ULK1, TP53, Autophagy receptor

## Abstract

TRIM proteins are characterized by their conserved N-terminal RING, B-box, and coiled-coil domains. These proteins are efficient regulators of autophagy, apoptosis, and innate immune responses and confer immunity against viruses and bacteria. TRIMs function as receptors or scaffold proteins that target substrates for autophagy-mediated degradation. Most TRIMs interact with the BECN1-ULK1 complex to form TRIMosomes, thereby efficiently targeting substrates to autophagosomes. They regulate the functions of ATG proteins through physical interactions or ubiquitination. TRIMs affect the lipidation of MAP1LC3B1 to form MAP1LC3B2, which is a prerequisite for phagophore and autophagosome formation. In addition, they regulate MTOR kinase and TFEB, thereby regulating the expression of ATG genes. TRIM proteins are efficient regulators of apoptosis and are crucial for regulating cell proliferation and tumor formation. Many TRIM proteins regulate intrinsic and extrinsic apoptosis via the cell surface receptors TGFBR2, TNFRSF1A, and FAS. Mitochondria modulate the anti- and proapoptotic functions of BCL2, BAX, BAK1, and CYCS. These proteins use a multipronged approach to regulate the intrinsic and extrinsic apoptotic pathways, culminating in coordinated activation or inhibition of the initiator and executor CASPs. Furthermore, TRIMs can have a dual effect in determining cell fate and are therefore crucial for cellular homeostasis. In this review, we discuss mechanistic insights into the role of TRIM proteins in regulating autophagy and apoptosis, which can be used to better understand cellular physiology. These findings can be used to develop therapeutic interventions to prevent or treat multiple genetic and infectious diseases.

**Graphical Abstract:**

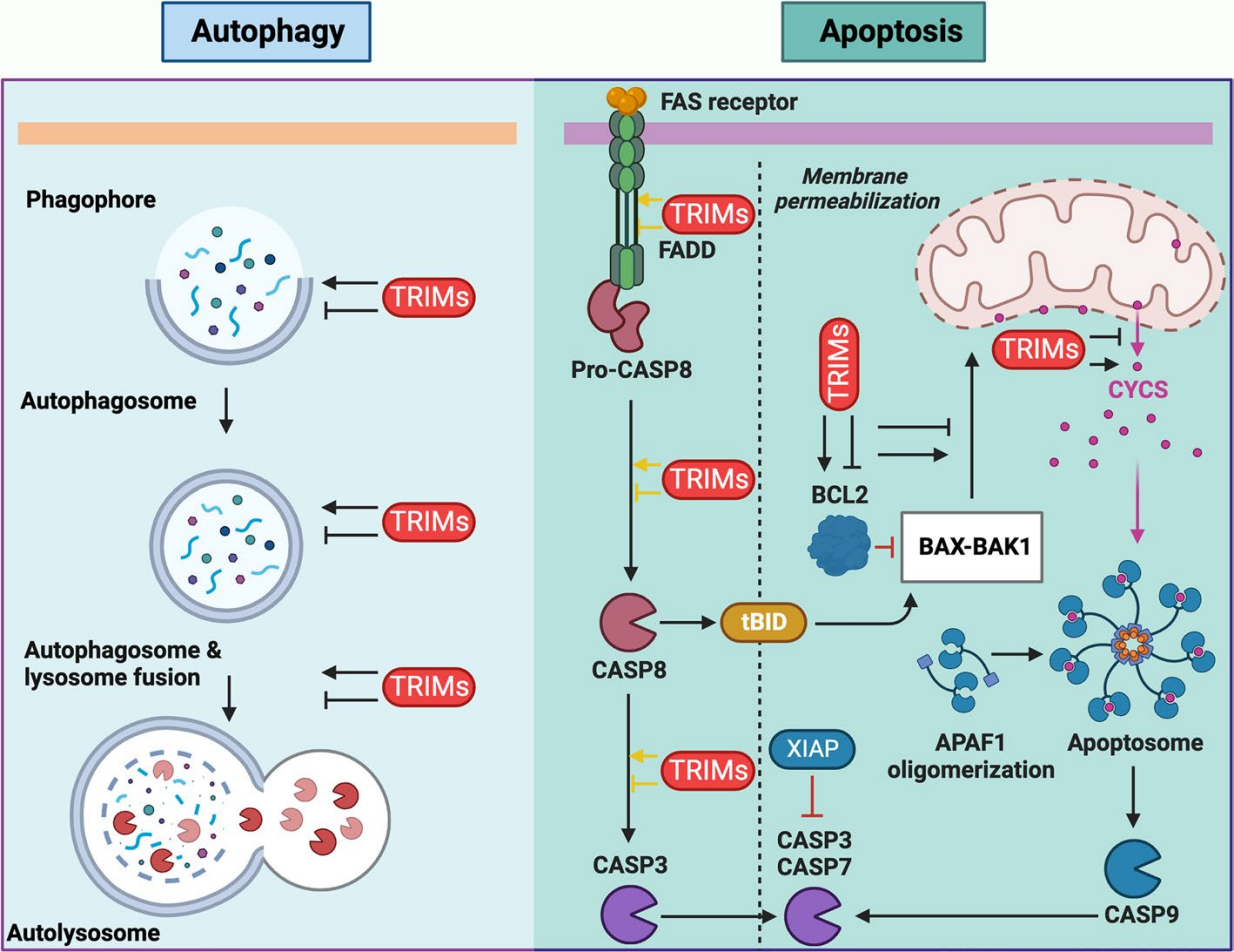

## Introduction

**Fig. 1:**
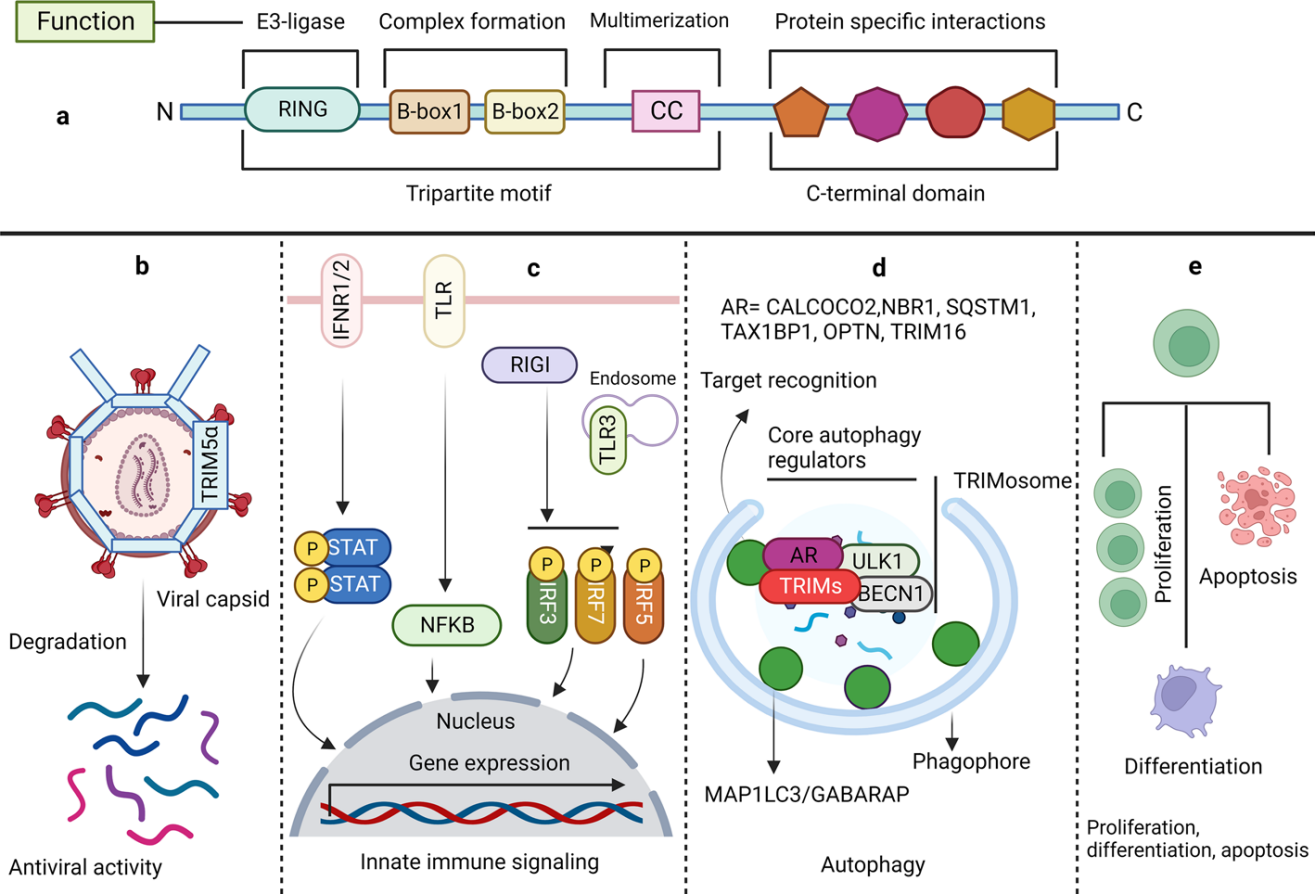
TRIM proteins possess a unique RBCC domain at their N-terminus and regulate various biological processes. **a** Domain organization of TRIM proteins at the N- and C-termini. The N-terminus contains a conserved RBCC domain, whereas the C-terminus contains variable domains required for protein‒protein interactions and substrate selection. **b** TRIM5α provides immunity against retroviruses, including HIV-1. It forms a hexagonal ring around the capsid, preventing the release of the genome and targeting the autophagosome-mediated degradation of HIV-1. **c** TRIM proteins regulate signaling events emanating from IFN receptors, TLRs, and RIGI receptors and activate IFN and NFKB signaling to produce proinflammatory cytokines. **d** TRIMs interact with the core components of autophagy, such as the BECN1-ULK1 autophagy-initiating complex and Ub-interacting autophagy receptors, to efficiently target substrates to autophagosomes for lysosome-mediated degradation. **e** TRIM proteins are essential regulators of cell proliferation, differentiation, and apoptosis. TRIM; Tripartite motif containing, RBCC; RING, B-box, Coiled-coil, TRIM5α; Tripartite motif containing 5α, HIV-1, Human immune deficiency virus-1, IFN; Interferon, TLR; Toll-like receptor, TLR3; Toll-like receptor 3, RIGI; RNA sensor RIG-I, NFKB; Nuclear factor kappa B, BECN1; Beclin 1, ULK1; Unc-51 like autophagy activating kinase 1, TLR3; Toll-like receptor 3, RING; Really interesting new gene, IFNR; Interferon production regulator, IRF3; Interferon regulatory factor 3, IRF5; Interferon regulatory factor 5, IRF7; Interferon regulatory factor 7, AR; Autophagy receptor, CALCOCO2; Calcium binding and coiled-coil domain 2, NBR1; NBR1 autophagy cargo receptor, SQSTM1; Sequestome 1, TAX1BP1; Tax1 binding protein 1, OPTN; Optineurin, TRIM16; Tripartite motif containing 16, MAP1LC3; Microtubule associated protein 1 light chain 3, GABARAP; GABA type A receptor-associated protein, STAT; Signal transducer and activator of transcription

Cell death is an essential aspect of the efficient functioning of an organism. Various mechanisms maintain cellular homeostasis, as cell survival and death pathways communicate with each other to determine cell fate. Apoptosis and autophagy are two biochemically and morphologically distinct processes that regulate homeostasis [1–3]. Apoptosis, or programmed cell death (PCD), is the cell's decision to die, whereas autophagy or self-feeding is an attempt to survive [4, 5]. In some cases, autophagy can also lead to cell death, which is often referred to as "autophagic cell death." However, it is important to note that cell death by apoptosis is more widely accepted and recognized than that by autophagy [6, 7]. Both basal- and stress-induced mechanisms of autophagy and apoptosis are involved in the proper functioning of organisms. Several stressors and effectors, including tripartite motif-containing (TRIM) proteins, simultaneously regulate these processes (Fig. 1). Numerous proteins communicate through molecular signaling pathways to determine cell fate. In addition to autophagy and apoptosis, necrosis leads to unprogrammed cell death during cellular emergencies such as infection, toxic attack, and cancer [8, 9]. Autophagy and apoptosis are integral parts of a large physiological system that plays critical roles in an organism’s development, immunity, adaptation, and aging. Disturbances in the apoptotic and autophagic machinery can lead to various pathological diseases, such as liver disease, diabetes, neurodegeneration, infectious diseases, cardiomyopathy, autoimmune diseases, and cancer. Therefore, a deeper understanding of both processes is crucial for efficient treatment of these diseases [4, 8, 10].

TRIM proteins, marked by their characteristic amino terminus-bearing RBCC domains (consisting of RING, B-Box1/2, and coiled-coil structures), exhibit notable diversity in their functional domains. These RBCC domains, either independently or in conjunction with various carboxy-terminal domains (CTDs), categorize TRIM proteins into 13 distinct subtypes **(**Fig. 1a**)**. The functional roles of TRIM proteins in autophagy and apoptosis, along with their corresponding domains, are detailed in Table 1 (Fig. 1a) [11, 12]. The RING domain within TRIM proteins encompasses a Zn2 + finger and exerts E3 ubiquitin (Ub) ligase activity, facilitating the conjugation of Ub to specific substrates or the protein itself via autoubiquitination [13]. The N-terminal B-boxes, although less functionally characterized, are presumed to contribute to the formation of large protein complexes [14]. Coiled coils (CCs) are instrumental in promoting the multimerization and assembly of higher-order oligomers. Notably, specific CCs feature an LC3-interacting region (LIR) motif, which is crucial for interactions with autophagy receptors. CTD plays a pivotal role in substrate diversity and recognition Fig. 1a [15, 16]. For example, TRIM5α is a pivotal player in cellular defense against viral infections. By leveraging its tripartite motifs, TRIM5α forms higher-order multimers, effectively creating a hexagonal mesh around viral capsids. This unique structure impedes the release of viral genomes and augments the degradation of viral components via autophagosome-mediated pathways (Fig. 1b). Moreover, TRIM proteins orchestrate innate immune responses by modulating downstream signaling initiated by Toll-like receptors (TLRs), RIGI-like receptors (RLRs), NOD-like receptors (NLRs), and the cyclic GMP-AMP synthase-stimulator of interferon response cGAMP interactor 1 (CGAS-STING1) (Fig. 1c) [17, 18]. The E3-Ub ligase activity of the RING domains extends beyond ubiquitination and encompasses the sumoylation or ISGylation of TRIMs and other proteins, thereby regulating various signaling events. A significant proportion of TRIM proteins exhibit responsiveness to interferons (IFNs), functioning as downstream effectors that govern immune responses to viral infections, including retroviruses. TRIM proteins such as TRIM5α, TRIM22, TRIM32, and TRIM15 have been implicated in the inhibition of the human immunodeficiency virus (HIV) life cycle by regulating viral integration, transcription, and assembly. Additionally, TRIM proteins such as TRIM21, TRIM25, TRIM27, TRIM30, and TRIM32 operate downstream of IFNs and pathogen recognition receptors, regulating immune responses to both bacterial and viral infections by activating key transcription factors like IFN regulatory factors and nuclear factor KB (NFKB) (Fig. 1c) [13, 18–20]. Furthermore, TRIM21, through its B-box 30.2, binds to the constant region of immunoglobulin G (IgG) in the cytoplasm, triggering opsonization and augmenting xenophagy [21, 22]. These diverse functionalities and intricate interactions highlight the multifaceted roles of TRIM proteins in cellular processes, particularly immune responses and antiviral defense mechanisms.

**Table 1:** Structural features of TRIM proteins involved in autophagy and apoptosis

Autophagy	Apoptosis	Autophagy + Apoptosis	Classification	Domains [N–C]
	TRIM1		C-I-2	R-B1-B2-CC-COS-FN3-SPRY
TRIM63			C-II	R-B2-CC-COS-ACID
TRIM6, TRIM11, TRIM50	TRIM27	TRIM17, TRIM20, TRIM21, TRIM35, TRIM39, TRIM72	C-IV-1	R-B2-CC-PRY-SPRY
TRIM5	TRIM34	TRIM22	C-IV-2	R-B2-CC-SPRY
	TRIM69		C-IV-4	R-CC-PRY-SPRY
	TRIM14	TRIM16	C-IV-4?	B2-CC-PRY-SPRY
TRIM49	TRIM48		C-IV-5	R-B2-SPRY
	TRIM8, TRIM19		C-V-1	R-B1-B2-CC
TRIM31			C-V-2	R-B2-CC
		TRIM28	C-VI	R-B1-B2-CC-PHD-BROMO
		TRIM32	C-VII-3	R-B2-CC-NHL
		TRIM37	C-VIII	R-B2-CC-MATH
TRIM23			C-IX	R-B1-B2CC-ARF
TRIM59		TRIM13	C-XI	R-B2-CC-TM

Most TRIM proteins contain a RING-finger domain, one or two B-box (B1 or B2) domains, and a coiled-coil domain

TRIM proteins were classified as C-I to C-XI. TRIM proteins without a RING finger domain (no RING) were unclassified

R, RING-finger domain; B1, B-box domain 1; B2, B-box domain 2; CC, coiled-coil domain; COS, cos box; FN3, fibronectin type III repeat; PRY, PRY domain; SPRY, SPRY domain; ACID, acid-rich region; NHL, NHL domain; PHD, PHD domain; BROMO, bromodomain; MATH, Meprin and TRAF-homology domain; ARF, ADP-ribosylation factor family domain; TM, transmembrane domain. TRIM; Tripartite motif containing, RING, Really interesting new gene, C-I; Class I, N–C; N-terminal-C-terminal domain

Current evidence underscores the pivotal role of TRIM proteins in the regulation of apoptosis, which significantly influences cellular fate [23]. Among these, TRIM19, which is prominently involved in acute promyelocytic leukemia (APL), plays a crucial role in controlling cell growth and tumorigenesis. *TRIM19*-lacking mice and primary cells have been observed to be rescued from stimulus-induced apoptosis, indicating the protein's inhibitory role in apoptotic pathways. The loss of *TRIM19* in APL confers a potential survival advantage to leukemic cells, resulting in the increased proliferation of tumor cells [24, 25]. Similarly, the truncated form of TRIM20 has been associated with impaired macrophage apoptosis in a familial Mediterranean fever model [26]. Additionally, in vitro studies have highlighted the proapoptotic activity of TRIM proteins such as TRIM32 and TRIM35 [27–29]. Autophagy, characterized by its remarkable selectivity in targeting cargo for autophagosomes, involves autophagy receptors that facilitate the linkage of ubiquitinated substrates to autophagosomes. These receptors establish connections through interactions with the microtubule-associated protein 1 light chain 3 (MAP1LC3) family of proteins that bear the LIR motif [30]. Their expression on a target or interaction with its components relies on various posttranslational modifications, among which ubiquitination plays a prominent role [31, 32]. Ubiquitination is an example of a post-translational modification that recruits autophagic receptors during cargo selection [33, 34]. A group of proteins, including sequestosome 1 (SQSTM1/p62), NBR1 autophagy cargo receptor (NBR1), optineurin (OPTN), calcium-binding and coiled-coil domain 2 (CALCOCO2), and Tax1 binding protein 1 (TAX1BP1), which harbor both the LIR-motif and Ub-binding domains, recognize Ub [30]. These autophagy receptors mediate the degradation of ubiquitinated substrates, including protein aggregates (SQSTM1, NBR1, and OPTN), dysfunctional organelles (OPTN, CALCOCO2, TAXBP1, and NBR1), and intracellular pathogens (SQSTM1, OPTN, CALCOCO2, TAXBP1, and NBR1) (Fig. 1d) [30, 35]. In this regulatory landscape, TRIM proteins are critical players in the modulation of core autophagy-related protein (ATG) functions. They contribute by either providing stability to ATG proteins or utilizing Ub-mediated mechanisms [36]. Moreover, TRIM proteins demonstrate exceptional selectivity in identifying pathogens, directing them to autophagosomes for degradation and enhancing the stability of signaling regulators involved in the autophagic process [37, 38].

This orchestrated interplay reflects the multifaceted roles of TRIM proteins in both apoptotic regulation and highly selective autophagic processes, indicating their significance in cellular homeostasis and immune response modulation (Fig. 1a-e). Understanding the function of TRIM proteins in the regulation of autophagy and apoptosis will broaden our knowledge of human diseases and cell proliferation.

In this review, we have thoroughly examined and synthesized crucial insights into the physiological and pathophysiological roles of TRIM proteins in the regulation of apoptosis and autophagy. Analysis of the literature and research findings in this field provides a valuable molecular understanding of the implications of TRIM proteins, which could have significant relevance for the design of therapeutic interventions (Fig. 1).

## Ub as a key player in autophagy-mediated cargo degradation

**Fig. 2:**
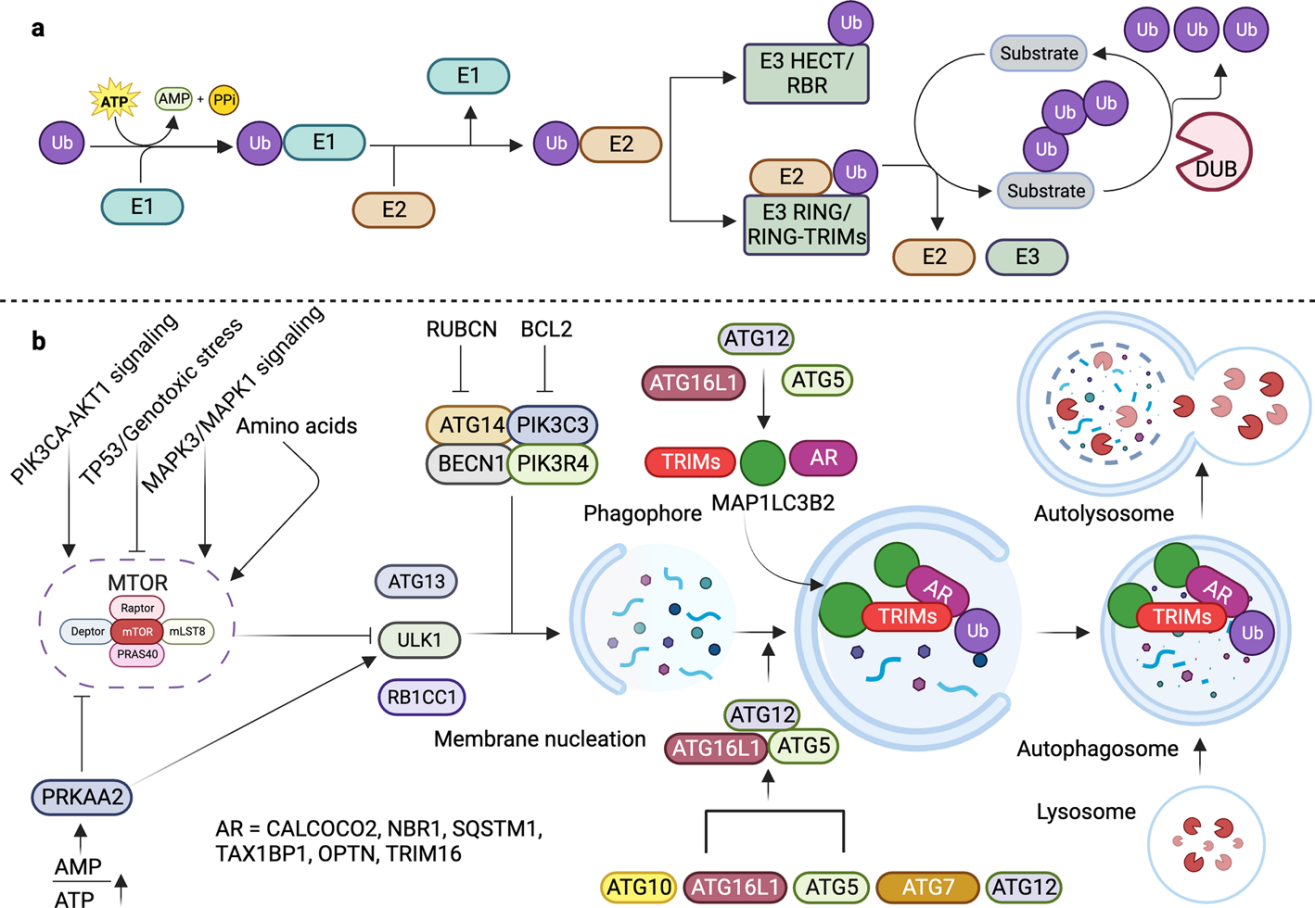
RING domain-containing TRIMs act as E3-Ub ligase and conjugate Ub to the target substrate, regulating autophagy. **a** Three enzymes catalyze the transfer of Ub to the target substrate. E1 catalyzes the conversion of Ub to E2 via ATP hydrolysis. E3-Ub ligases containing RING domains directly transfer Ub to the target substrate, whereas HECT and RBR E3-Ub ligases form E3-Ub intermediates before transferring Ub to the target substrates. **b** Various signal inputs converge on the main autophagy regulator MTOR, such as PIK3CA, TP53, MAPK3/MAPK1, hunger, and PRKAA2/AMPK. Activation of the MTOR kinase complex inhibits autophagy by inducing inhibitory phosphorylation of the autophagy-initiating kinase ULK1. PRKAA2 activated autophagy by phosphorylating ULK1. TRIM proteins regulate various steps of canonical autophagy, including autophagy initiation, phagophore formation, MAP1LC3 conjugation, and efficient targeting of substrates to autophagosomes via autophagy receptors and Ub. Autophagosomes fuse with lysosomes to form autolysosomes, which degrade the entrapped cargo materials. TRIM; Tripartite motif containing, Ub; Ubiquitin, ATP; Adenosine triphosphate, RING; Really interesting new gene, HECT; Homologous to the E6-AP carboxyl terminus, RBR; RING-in-between-RING, MTOR; Mechanistic target of rapamycin kinase, PIK3CA; Phosphatidylinositol-4,5-bisphosphate 3-kinase catalytic subunit alpha, TP53; Tumor protein 53, MAPK3/MAPK1; mitogen-activated protein kinase 3/1, PRKAA2/AMPK; Protein kinase AMP-activated catalytic subunit alpha 2, ULK1; Unc-51 like autophagy activating kinase 1, MAP1LC3B2; Microtubule associated protein 1 light chain 3 beta 2, AKT1; AKT serine/threonine kinase 1, PPi; Inorganic pyrophosphate, AMP; Adenosine monophosphate, RUBCN; rubicon autophagy regulator, BCL2; BCL2 apoptosis regulator, ATG13; Autophagy related 13, RB1CC1; RB1 inducible coiled-coil 1, ATG10; Autophagy related 10, ATG7; Autophagy related 7, ATG12; Autophagy related 12, ATG14; Autophagy related 14, PIK3C3; Phosphatidylinositol 3-kinase catalytic subunit type 3, PIK3R4; phosphoinositide-3-kinase regulatory subunit 4, BECN1; Beclin 1, ATG16L1; Autophagy related 16 like 1, ATG5; Autophagy related 5, AR; Autophagy receptor, CALCOCO2; Calcium binding and coiled-coil domain 2, NBR1; NBR1 autophagy cargo receptor, SQSTM1; Sequestome 1, TAX1BP1; Tax1 binding protein 1, OPTN; Optineurin, TRIM16; Tripartite motif containing 16, ATG10; Autophagy related 10, ATG7; Autophagy related 7, ATG12; Autophagy related 12, ATG13; Autophagy related 13, DUB; Deubiquitinase

Autophagy is an indispensable physiological process that maintains cellular homeostasis in response to various cellular stresses such as starvation or pathogen infestation. It orchestrates the degradation and recycling of cellular components entrapped within autophagosomes, encompassing damaged organelles, misfolded protein aggregates, and intracellular pathogens [39–41]. While primarily offering energy during nutrient deprivation, autophagy also exhibits a dual nature by not only degrading cellular contents, but also triggering cell secretion [42, 43]. The bidirectional effects of autophagy are beneficial under normal conditions and detrimental under dysregulated conditions. They are notably associated with an array of pathological conditions such as cancer, neurodegenerative disorders, and lysosomal malfunctions [10, 44]. Although traditionally considered nonselective, recent observations have unveiled the role of selectivity in autophagy, particularly in targeting intracellular pathogens through Ub and autophagy receptors for autophagosome-mediated degradation. Ubiquitination, a pivotal post-translational modification process, plays a central role in cellular protein degradation via the Ub-proteasome system (UPS) (Fig. 2a) [45–47].

Ub, a 76 amino acid conserved protein present in all eukaryotes, is covalently conjugated to the target proteins via an isopeptide bond [48]. The C-terminal glycine of Ub and ε-amino group of lysine on substrates are involved in covalent isopeptide bond formation. Ubiquitination can occur in a variety of ways, including mono-, di-, or polyubiquitination, which is often referred to as the “Ub code” [49]. This code determines the fate and alters the biological functions of substrate proteins. The diverse topology of Ub linkages enables the transmission of the complex physiological signals required for spatiotemporally controlled cellular functions. K11- and K48-linked poly-Ub chains adopt compact structures that direct substrates to the canonical Ub-proteasome pathway for degradation [30]. However, when Ub is attached via Met1 or K63 linkages, these adducts adopt an extended conformation, enabling reversible recruitment of multi-protein complexes. These complexes are key non-proteolytic consequences of the Ub conjugation events found in immune signaling cascades [30, 50]. Although previous research has primarily focused on homogenously linked Ub chains, heterogeneously branched Ub chains have emerged as crucial protein modifications. These post-translational modifications involving Ub further expand the specificity, versatility, and efficacy of Ub-dependent signaling events [51]. Protein ubiquitination is a three-step enzymatically catalyzed, reversible reaction. E1, a Ub-activating enzyme, catalyses the formation of a high-energy thioester intermediate using the C-terminal glycine of Ub and its active site cysteine in an Mg2 + -dependent manner. Thereafter, a Ub-conjugating (E2) enzyme's active site, cysteine, accepts activated Ub from charged E1. Finally, an E3-Ub ligase interacts with charged E2, the substrate protein, and Ub, leading to the formation of the Ub adduct with the target protein [52, 53]. Similar to other post-translational modifications, it requires a specialized class of proteases, known as deubiquitinating enzymes, to remove Ub (Fig. 2a) [54]. While the UPS and autophagy pathways operate independently through lysosomal and proteasomal degradation, a significant overlap exists between these systems. Ubiquitination confers substantial selectivity for substrate recognition during autophagy, emphasizing the critical involvement of various factors engaged in Ub conjugation, including E3 ligases and the thirty-five identified ATG proteins (Fig. 2) [55–57].

The mechanistic target of rapamycin kinase (MTOR) is a major regulator of autophagy in mammalian cells and actively modulates autophagy pathways in response to nutrient availability. Upon activation in nutrient-rich conditions, MTOR impedes autophagy by inhibiting the formation of active ATG protein complexes. [58–60]. Conversely, nutrient deprivation suppresses MTOR activity, fostering the upregulation and activation of autophagy pathways. MTOR exerts its inhibitory effect by phosphorylating key autophagy-related proteins, such as unc-51-like autophagy-activating kinase 1 (ULK1), hindering its activation and function. This phosphorylation, particularly at serine 757, inhibits the activation and translocation of ULK1 and regulates autophagy initiation. It also prevents phosphorylation at multiple sites, including serine 555, which is responsible for activation, protein–protein interaction, and translocation into the mitochondria by protein kinase AMP-activated catalytic subunit alpha 2 (PRKAA2/AMPK) (Fig. 2b) [61–64].

Additionally, TRIM proteins, functioning as Ub ligases, significantly affect critical autophagy regulators and receptors, adding another layer of complexity to the regulation of autophagy. The association of the TRIM family with BECN1 and ULK1 highlights an intricate regulatory network governing autophagy. Through their Ub ligase activity, TRIM proteins potentially modulate the function and activity of BECN1 and ULK1, further emphasizing their role in the selectivity and regulation of autophagy.

## TRIM proteins in bulk autophagy: masters of BECN1 and ULK1 complex regulation

The TRIM family of RING-E3 ligases serves as crucial regulators of autophagy and its receptors. Mandell et al. performed systematic screening to determine the involvement of TRIM proteins in autophagy and reported their regulatory functions. TRIM proteins act as regulators, triggering the activation of the autophagy-initiating kinase ULK1 and autophagy regulator BECN1. These results indicated that TRIMs facilitate the formation of multimolecular complexes with ULK1 and BECN1, known as TRIMosomes (Figs. 1 and 2b) [65, 66]. In the study by Mandell et al., several TRIMs have been suggested to interact with ATG proteins to regulate autophagy (e.g. TRIMs 5α, 6, 16, 17, 20) [67]. Furthermore, another study found that TRIM20 and TRIM21 orchestrate ULK1, BECN1 and autophagy-related 16-like 1 (ATG16L1) and consequently interact with the mammalian homologs of ATG8 proteins, preferring the autophagosome-lysosome fusion protein GABA type A receptor-associated protein (GABARAP) (Figs. 1 and 2b) [68, 69].

**Fig. 3:**
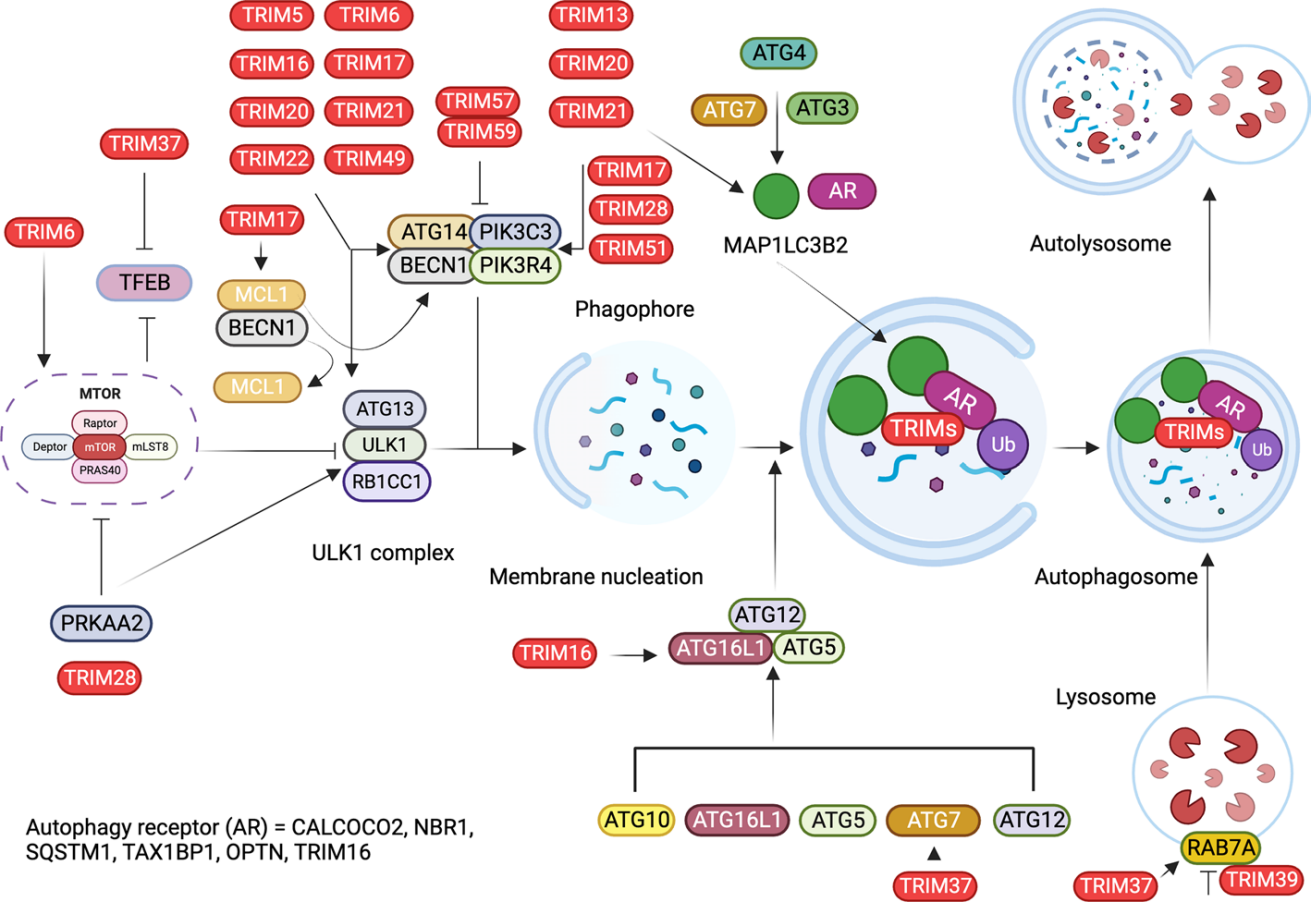
TRIM proteins regulate autophagy by modulating the MTOR complex, TFEB, ULK1-BECN1 complex, and MAP1LC3 conjugation. TRIM proteins regulate autophagy at various steps, including the activity of MTOR, TFEB, the BECN1-ULK1 complex, and the conjugation of MAP1LC3 to form MAP1LC3I and MAP1LC3II to form phagophores and autophagosomes. Autophagosomes fuse with lysosomes to form autolysosomes, which breakdown and recycle cargo materials and autophagosomes. Autophagosomes fuse with lysosomes to form autolysosomes, degrade cargo materials, and recycle. MTOR; Mechanistic target of rapamycin kinase, TFEB; Transcription factor EB, ULK1; Unc-51 like autophagy activating kinase 1, BECN1; Beclin 1, MAP1LC3B2; Microtubule associated protein 1 light chain beta 2, TRIM; Tripartite motif containing, Ub; Ubiquitin, AR; Autophagy receptor, CALCOCO2; Calcium binding and coiled-coil domain 2, NBR1; NBR1 autophagy cargo receptor, SQSTM1; Sequestome 1, TAX1BP1; Tax1 binding protein 1, OPTN; Optineurin, TRIM16; Tripartite motif containing 16, Autophagy related 14, PIK3C3; Phosphatidylinositol 3-kinase catalytic subunit type 3, PIK3R4; phosphoinositide-3-kinase regulatory subunit 4, ATG5; Autophagy related 5, ATG10; Autophagy related 10, ATG7; Autophagy related 7, ATG12; Autophagy related 12, ATG13; Autophagy related 13, ATG4; Autophagy related 4, ATG3; Autophagy related 3, RB1CC1; RB1 inducible coiled-coil 1, RAB7A; RAB7A, member RAS oncogene family

ULK1 is a key protein kinase that is part of a larger protein complex called the ULK complex. This complex is essential for the induction of autophagy in response to cellular stress, nutrient deprivation, or other signals that prompt the need for autophagy. Upon activation, ULK1 phosphorylates proteins such as the lipid kinase PI3KC3, which is essential for autophagosome biogenesis. ULK1 also interacts with and phosphorylates other key autophagy-related proteins, such as ATG14 and BECN1, further contributing to the regulation of autophagosome formation [70]. BECN1 is a vital protein that acts as the central regulator for the assembly of components of the BECN1-phosphatidylinositol 3-kinase catalytic subunit type 3 (PIK3C3) and phosphoinositide 3-kinase regulatory subunit 4 (PIK3R4) complex to activate autophagy (Figs. 1 and 2b). Various BECN1 regulators have been identified. For example, in non-small cell lung cancer, TRIM59 negatively regulated NFKB signaling and affected BECN1 transcription (Fig. 3). It regulates tumor necrosis factor receptor-associated factor 6 (TRAF6)-mediated K63-linked ubiquitination of BECN1, thereby modulating the formation of the BECN1-PIK3C3 complex. TRIM59 also activates K48-linked ubiquitination and degradation of BECN1. Furthermore, these results indicate that TRIM59 is a dual regulator of BECN1, which affects transcription and ubiquitination [71]. Similarly, TRIM50-mediated K63-linked ubiquitination of BECN1 has been reported. TRIM50 promotes autophagy by ubiquitinating BECN1 at lysine 372, facilitating its interaction with ULK1. Lysine 372 of TRIM50 is critical for acetylation and is therefore a target of histone deacetylase 6 (HDAC6) [72]. In particular, HDAC6 supports the formation of aggresomes, which are a collection of microtubule-associated polyubiquitinated proteins. TRIM50 interacts with HDAC6 and SQSTM1 to regulate autophagic clearance of aggresomes [73].

TRIMs such as TRIM17 play a dual role in regulating autophagy. TRIM17 downregulates autophagy by inhibiting BECN1 and stabilizing the anti-autophagy protein MCL1 apoptosis regulator BCL2 family member (MCL1). Conversely, TRIM17 removed MCL1 from midbodies containing MCL1-BECN1 complexes, thereby dismantling them and activating autophagy (Fig. 3) [74]. TRIM28 functions as both a pro-autophagy and anti-autophagy factor under different circumstances. This dichotomy is the result of the coordinated actions of multiple factors (Fig. 3) [75].

## TRIM proteins in selective autophagy

### Autophagy-mediated defense against viral pathogenesis

Inflammasomes are multiprotein complexes that play a critical role in the innate immune response of the body by activating an inflammatory response. The core components of an inflammasome typically include a cytoplasmic pattern recognition receptor (PRR), an adaptor protein, and an effector protein known as caspase 1 (CASP1) [76]. PRRs, such as NLRs (Nod-like receptors) or AIM2 (Absent in Melanoma 2), are responsible for detecting various pathogen-associated molecular patterns (PAMPs) or danger-associated molecular patterns (DAMPs) [77]. Inflammasome activation is a tightly regulated process, as excessive or dysregulated inflammation can lead to tissue damage and chronic inflammatory diseases. Autophagy acts as a control mechanism for inflammasome activation. By clearing away potential activators, autophagy helps prevent excessive or inappropriate inflammation. Additionally, autophagy can directly target and degrade inflammasome components themselves, thereby limiting their activity [78, 79]. Several TRIM proteins influence inflammasome activation by regulating the turnover of inflammasome components through autophagy. As autophagy is closely associated with the development of an inflammatory response, the function of TRIMs as receptors has been studied extensively [80, 81].

**Fig. 4:**
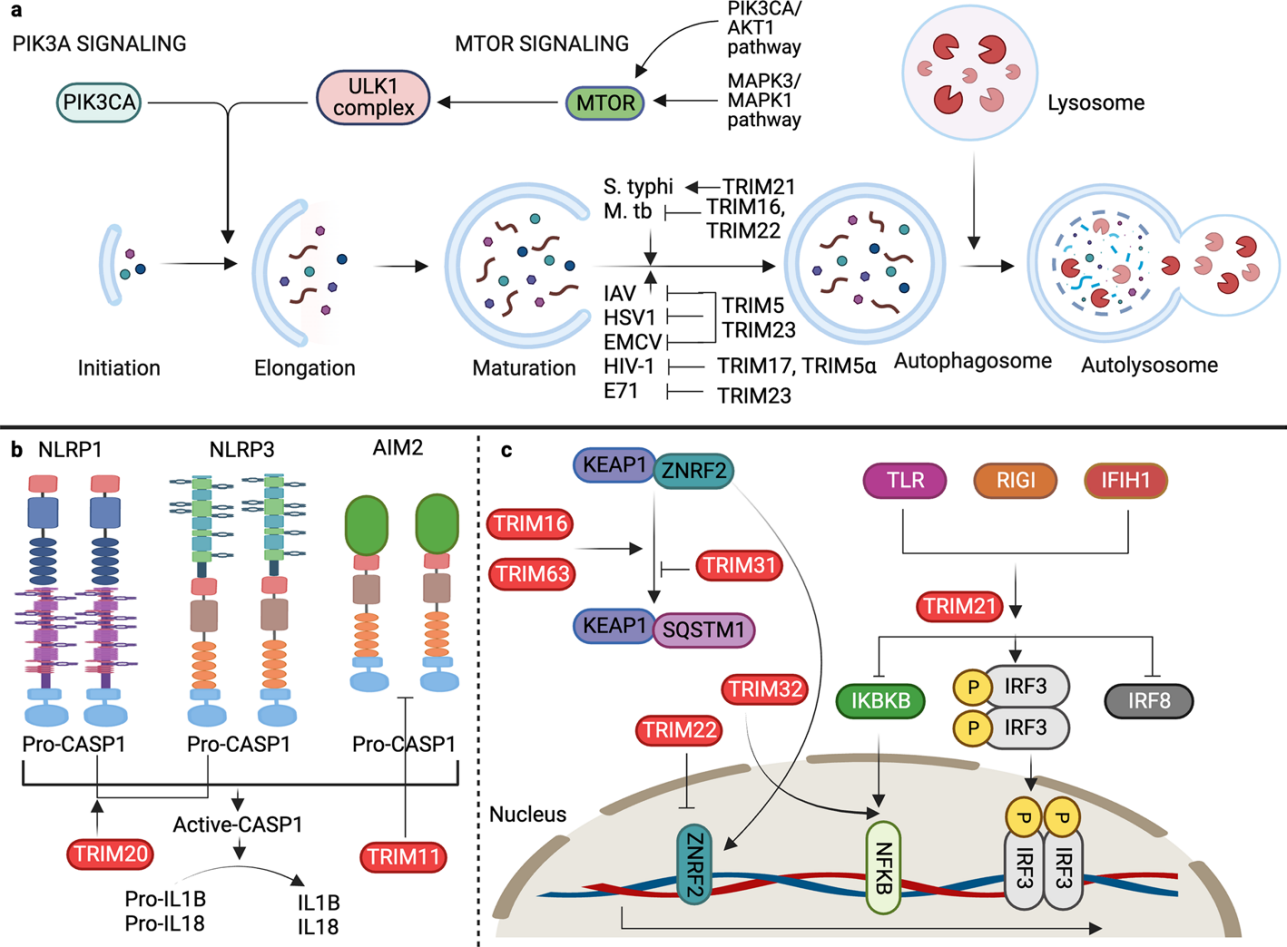
TRIM protein-mediated autophagy is involved in regulating immunity against various bacteria and viruses. **a** Autophagy involves the initiation of phagophores regulated by ULK1. Class III phosphatidylinositol kinases, the ULK1-BECN1 complex, and MTOR kinase regulate the initiation of autophagy and the formation of phagophores. MTOR activity is regulated by PRKAA2, PIK3CA-AKT1 signaling, and MAPK3/MAPK1 signaling. The phagophores mature into autophagosomes and fuse with lysosomes to form autolysosomes. TRIM proteins target bacteria and viruses to autophagosomes to degrade and activate innate immune signaling. **b** TRIM proteins activate inflammasome complexes and convert pro-CASP1 to active CASP1, producing the proinflammatory cytokines IL1B and IL18. **c** TRIMs regulate the KEAP1-SQSTM1-ZNRF2 signaling axis and ZNFR2-mediated transcription. TRIM proteins also regulate IFN and NFKB signaling cascades to induce the production of proinflammatory cytokines. TRIM; Tripartite motif containing, ULK1; Unc-51 like autophagy activating kinase 1, BECN1; Beclin 1, MTOR; Mechanistic target of rapamycin kinase, PRKAA2; Protein kinase AMP-activated catalytic subunit alpha 2, PIK3CA; Phosphatidylinositol-4,5-bisphosphate 3-kinase catalytic subunit alpha, MAPK3/1; Mitogen-activated protein kinase 3/1, CASP; Caspase, IL1B; Interleukin 1 beta, IL18; Interleukin 18, KEAP1; Kelch like ECH associated protein 1, SQSTM1; Sequestome 1, ZNRF2; Zinc and ring finger 2, IFN; Interferon, NFKB; Nuclear factor kappa B, AKT1; AKT serine/threonine kinase 1, S. typhi; Salmonella typhi, M. tb; Mycobacterium tuberculosis, IAV; Influenza A virus, HSV1; Herpes simplex virus 1, EMCV; Encephalomyocarditis virus, HIV-1; Human immune deficiency virus, E71, Enterovirus 71, NLRP1; NLR family pyrin domain containing 1, NLRP3; NLR family pyrin domain containing 3, AIM2; Absent in melanoma 2, CASP1; Caspase 1, TLR; Toll-like receptor, RIGI; RNA sensor RIG-I, IFIH1; Interferon induced with helicase C domain 1, IKBKB; Inhibitor of nuclear factor kappa B kinase subunit beta, IRF3; Interferon regulatory factor 3, IRF8; Interferon regulatory factor 8

TRIM5α has been shown to act as a receptor for HIV-1 capsid protein p24, providing it for autophagic degradation (Figs. 1b and 4a). TRIM5α forms cytoplasmic bodies that serve as signaling complexes that play a critical role in retroviral restriction. Additionally, nonsignaling aggregates of TRIM5α are involved in retroviral restriction [82]. TRIM11 has been shown to negatively regulate the inflammasome complex AIM2 (Fig. 4b). AIM2 is activated by bacterial and viral double-stranded (ds) DNA in the cytosol and mediates inflammatory activity via interleukin 1 beta (IL1B) and interleukin 18 (IL18) (Fig. 4b). Activated TRIM11 binds to AIM2 and associates with SQSTM1 after autopolyubiquitination at lysine 458. SQSTM1 is a multifunctional autophagy receptor that acts as a link between autophagy and the UPS. SQSTM1 is degraded along with AIM2, thereby suppressing inflammasome activity [83]. Similarly, other TRIM proteins also function as receptors for inflammasome components, such as NLR family pyrin domain containing 3 (NLRP3), NLRP1, pro-caspase 1 (pro-CASP1), and IRF3 of the NLR family. TRIM20 targets and binds to NLRP3, NLRP1, and pro-CASP1, while TRIM21 associates with IRF3 (Fig. 4b). In response to TLR stimulation, the PRYSPRY domain of TRIM21 is phosphorylated and interacts with IRF3. IRF3 is then degraded, suppressing type 1 IFN production and thereby negatively regulating the innate immune response [84, 85].

*TRIM20* mutations lead to autoinflammatory diseases such as familial Mediterranean fever (FMF) [86]. Subsequently, both TRIM20 and TRIM21 target inflammasome components for autophagic degradation [89]. In addition to acting as a fragment crystallizable receptor (FcR) protein for IRF3, which acts as a positive regulator of autophagy, TRIM21 ubiquitinates and degrades interferon regulatory factor 8 (IRF8), a regulator of autophagy. Here, TRIM21 is a negative regulator, as it degrades IRF8, which is a positive regulator of autophagy, thereby providing context-dependent regulation of various autophagic processes [87]. TRIM21 monobiquitinates the inhibitor of nuclear factor kappa B kinase subunit beta (IKBKB), which mediates its autophagy-mediated degradation and thus affects NFKB signaling (Fig. 4c) [88]. TRIMs have also been reported to act as effectors of antiviral defense. TRIM5α and TRIM23 contribute to autophagy-mediated restriction of several viruses, including herpes simplex virus 1 (HSV-1), encephalomyocarditis virus (EMCV), and influenza A virus (IAV) (Fig. 4a). TRIM23 undergoes unusual K27-linked autoubiquitination of the ADP-ribosylation factor (ARF) domain, ultimately leading to dimerization and induction of TANK-binding kinase 1 (TBK1). This ubiquitination is followed by the phosphorylation of the selective autophagy receptor SQSTM1 and degradation of ubiquitinylated targets [89]. In contrast, TRIM19 plays a protective role against many DNA and RNA viruses by inhibiting autophagy. For example, in enterovirus 71 (EV71)-infected cells, virus-induced autophagy promotes EV71 proliferation, while TRIM19 opposes it. Therefore, several viral strains have evolved mechanisms to cleave TRIM19 using viral protease 3Cpro (Pro3C) [90]. Peng et al. demonstrated that TRIM32 from the crustacean *Penaeus monodon* plays a role in autophagy during white spot syndrome virus infection. The study found that TRIM32 upregulates NFKB, contributing to the regulation of the autophagic process in response to viral infection (Fig. 4c) [91].

### Regulation of bacterial pathogenesis

TRIMs are critical regulators of the innate and adaptive immune responses to bacterial invasion. TRIM22 restricts *Mycobacterium tuberculosis* (*M. tb*) infection and promotes the clearance of *M. tb* via the NFKB/BECN1-mediated autophagic pathway (Fig. 4a) [92]. TRIM21 is induced by interferon alpha 1 (IFNA1) and negatively regulates the innate immune response to *Salmonella typhimurium* (*S. typhimurium*) (Fig. 4a). TRIM21 is targeted by chaperone-mediated autophagy during infection [93]. It functions as an autophagy receptor in bacterial infections such as *M. tb* (Fig. 4a). TRIM16 works with galectins (LGALSs), specifically galectin 3 (LGALS3), to mobilize and assemble other autophagy regulators, such as ATG16L1 and BECN1, in a ULK1-dependent manner. LGALS3 senses membrane damage and recruits TRIM16 to regulate the MTOR-TFEB-mediated autophagy regulatory pathway, all of which play important roles in controlling *M. tb* infection [94]. The canonical autophagy pathways are regulated by ATG genes. Noncanonical autophagy pathways occur simultaneously with canonical autophagy within cells. These pathways are regulated by the formation of autophagosomes and autolysosomes even in the absence of ATG16L1, ATG5, and ATG7. A study by Ra et al. showed that normal autophagy clears the bacterial load in ATG5- and ATG7-deficient cells, suggesting the existence of alternative pathways. They also found that TRIM31, which is highly enriched in the mitochondria and gut, is downregulated in Crohn's disease. Furthermore, TRIM31 expression was inversely proportional to SQSTM1 levels, indicating TRIM31-mediated SQSTM1 degradation. TRIM31 separates from mitochondrial autophagic membranes and is enriched in the ATG5-ATG7-ATG16L1 complex. Further studies confirmed that even in the absence of ATG5 and ATG7, TRIM31 promotes autophagy through lipopolysaccharide (LPS) stimulation, which is the basis for bacterial clearance via an alternative pathway [95].

### Autophagy-mediated regulation of mitophagy and lysophagy

Damaged mitochondria with altered morphology, increased oxidative stress, and reduced ATP production are characteristic features of chronic kidney disease and associated skeletal atrophy [96]. While autophagy maintains renal homeostasis, mitophagy (selective autophagy of damaged mitochondria) maintains cellular homeostasis and muscle mass [97, 98]. TRIM27 has been shown to induce mitophagy by increasing mitochondrial aggregation. It interacts with TBK1, induces its interaction with SQSTM1, and recruits aggregated mitochondria to trigger mitophagy [99]. TRIM72 and autophagy and beclin 1 regulator 1 (AMBRA1) levels have been shown to be significantly low in patients with chronic kidney disease (CKD). Liu et al. (2020) demonstrated the protective role of TRIM72 against contrast agent-induced acute kidney injury by facilitating the reduction of cell membrane damage and apoptosis. TRIM72 migrates to the site of injury in renal proximal tubular cells and remains bound to phosphatidylserine to protect the cells [100]. Additionally, TRIM72 improves mitophagy and promotes the clearance of dysfunctional mitochondria by enhancing AMBRA1 expression, which may have therapeutic potential [101]. TRIM63 is a critical regulator of selective autophagy required for neuromuscular junction remodeling in fasting-induced skeletal muscle wasting. Autophagy, regulated by the autophagy receptor SQSTM1, leads to the formation of vesicles containing the membrane remodeler SH3 domain-containing GRB2-like endophilin B1 (SH3GLB1), TRIM63, and the essential cholinergic receptor (CHRN) (Fig. 4c). The process of increasing CHRN carrier levels, both under normal conditions and in response to atrophic stress, is dependent on TRIM63 [102]. Like MURF1/TRIM63, MURF2/TRIM55 is actively involved in the SQSTM1-dependent autophagic process. The two isoforms of TRIM55 protein, TRIM55A and TRIM55B, regulate the switch between autophagy and the Ub-proteasome system during differentiation of C2C12 muscle cells. The ratio of TRIM55A to TRIM55B isoforms changes during differentiation following sequential activation of autophagy or proteasomal degradation. TRIM55A and TRIM55B isoforms have different cellular locations and regulations in relation to SQSTM1, NBR1, and MAP1LC3. Emerging evidence suggests that TRIM55A may participate in both UPS and autophagic degradation, whereas TRIM55B is a novel MAP1LC3-interacting protein that is involved in autophagic vesicle formation. [103]. Araya et al. showed that TRIM16 is involved in selective lysophagy (autophagy-mediated breakdown of lysosomes) in human bronchial epithelial cells isolated from patients with chronic obstructive pulmonary disease (COPD). In patients with COPD, impaired autophagy leads to lysosomal membrane permeabilization. TRIM16 and LGALS3 act cooperatively to remove damaged lysosomes through lysophagy. This phenomenon is crucial for rescuing bronchial cells with lysosome-dependent dysregulated autophagy [104].

## Unraveling the crucial role of TRIM proteins in autophagy-driven cancer and neurodegenerative pathways

Autophagy plays a complex and dual role in cancer, having both protumorigenic and antitumorigenic effects depending on the stage and context. TRIMs, as regulators of autophagy, are critically intertwined, serving as proto-oncogenes or anti-oncogenes. MAGE family member A3 (MAGEA3 or 6) associates with TRIM28 to ubiquitinate PRKAA2 and degrade it. This results in inhibition of autophagy via the MTOR pathway and promotes tumorigenesis (Fig. 3). In the absence of MAGEA3 or 6, TRIM28 acts through the SUMOylation of PIK3C3, promoting BECN1 complex formation and regulating autophagy (Fig. 3). TRIM28 also contributes to glioblastoma pathogenesis by inducing autophagy [68, 69]. TRIM6 expression induces MTOR signaling by regulating the ubiquitination of its negative regulators, TSC complex subunits (TSC) 1 and 2, thus promoting renal fibrosis. (Fig. 3) [70]. TRIM22 inhibits osteosarcoma progression by promoting proteasomal degradation of zinc and ring finger 2 (ZNRF2) independent of goblet-like ECH-associated protein 1 (KEAP1), resulting in autophagic death of sarcoma cells [71]. TRIM39 plays a critical role in autophagy-mediated colon cancer progression. TRIM39 interacts with RAB7A and represses its activity by ubiquitination of lysine 191 to promote apoptosis (Fig. 3) [72]. In individuals with mulibrey nanism, a condition associated with tumor susceptibility, autophagy serves as a cell survival mechanism. Loss of TRIM37 induces autophagy in an MTORC1-dependent manner, serving as a pro-survival mechanism following the loss of TRIM37 (Fig. 3) [73, 74]. Under nutrient-rich conditions, TRIM37 interacts with MTOR and RRAGB proteins, enhancing the MTOR-RRAGB interaction and promoting lysosomal localization of MTORC1, thereby activating amino acid-stimulated MTORC1 signaling. TRIM37 loss leads to reduced TFEB phosphorylation and subsequent dissociation from its cytosolic chaperones tyrosine 3-monooxygenase/tryptophan 5-monooxygenase activation protein (YWHA), thereby translocates to the nucleus, and activates genes involved in lysosomal biogenesis and autophagy [75, 76]. TRIM37 depletion resulted in the inhibition of MTOR-TFEB signaling and induction of canonical autophagy mediated by ATG7 and ATG16L1 (Fig. 3) [73].

TRIMs also play a critical role in aggregate formation and clearance through autophagic degradation in diseases, such as neurodegeneration and cancer. Jena et al. (2018) reported that TRIM16 promotes protein aggregate formation in response to various stresses. This aggregate formation precedes their disassembly and disposal via the SQSTM1-KEAP1-ZNRF2 pathway (Fig. 4c). TRIM16 assembles key canonical autophagy mediators and channels their functions to promote aggregate turnover [77]. TRIM13, an endoplasmic reticulum (ER) resident Ub E3 ligase, regulates autophagy during ER stress. Through its coil domain, TRIM13 triggers autophagy, interacts with SQSTM1, and concurrently modulates ZNRF2 signaling. The induction of autophagy during ER stress significantly curtails the clonogenic ability of cells. Furthermore, in lung adenocarcinoma (LUAD), TRIM13 acts as a tumor suppressor by inhibiting cell proliferation and orchestrating autophagy via the KEAP1/ZNRF2 pathway. It mediates SQSTM1 ubiquitination and subsequent degradation, thereby negatively regulating ZNRF2 signaling and downstream antioxidants and ultimately promoting autophagy in LUAD cells [105–107]. These studies emphasize its dual significance in maintaining cellular equilibrium under stressful conditions and in specific cancer contexts. TRIM21 interacts with G3BP stress granule assembly factor 1 (G3BP1) and induces K63-linked ubiquitination. G3BP1 is a core component of the stress granules required for liquid‒liquid phase separation. TRIM21 cooperates with the autophagy receptors CALCOCO2 and SQSTM1 in the arsenate-induced stress response and plays an essential role in the removal of stress granules. These results suggest that TRIM21 and its induced autophagy are required for stress granule homeostasis [80].

## Embracing apoptosis: a complex pathway involving signaling proteins and CASPs

**Fig. 5:**
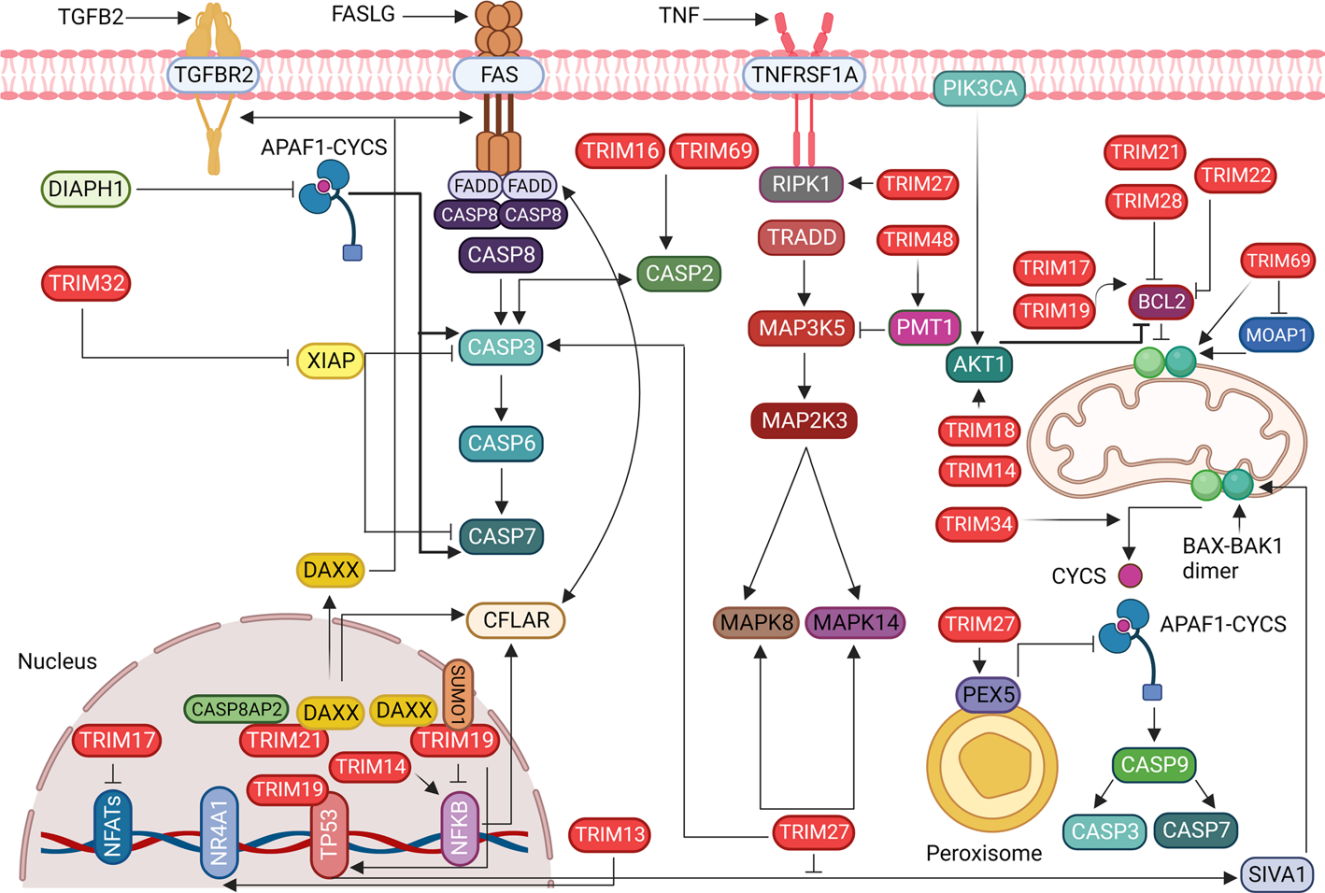
TRIM proteins regulate extrinsic and intrinsic apoptosis via diverse mechanisms. TRIMs regulate death receptor-mediated apoptosis by regulating the activation of initiator and executor CASPs and MAPK signaling cascades. TRIMs also regulate intrinsic apoptosis mediated by the mitochondria and the pro- and anti-apoptotic proteins BAX, BAK1, and BCL2. They induce the formation of mitochondrial pores to release CYCS, thereby inducing the formation of apoptosomes and the activation of CASP9 and CASP3. Some TRIMs activate anti-apoptotic BCL2 to inhibit mitochondrial pore formation and CYCS release, thereby preventing apoptosome formation and CASP9 activation. Several TRIM proteins are localized in the nucleus to regulate gene expression mediated by TP53, NFATs, and NFKB. NFKB; Nuclear factor kappa B, TGFB2; Transforming growth factor beta 2, FASLG; Fas ligand, TNF; Tumor necrosis factor, TGFBR2; Transforming growth factor beta receptor 2, FAS; Fas cell surface death receptor, TNFRSF1A; TNF receptor superfamily member 1A, PIK3CA; phosphatidylinositol-4,5-bisphosphate 3-kinase catalytic subunit alpha, DIAPH1; DIAPH1, APAF1; Apoptotic peptidase activating factor 1, DAXX; Death domain associated protein, NR4A1; Nuclear receptor subfamily 4 group A member 1, CASP8AP2; Caspase 8 associated protein 2, SUMO1; Small ubiquitin like modifier 1, FADD; Fas associated via death domain, CASP8; Caspase 8, CASP6; Caspase 6, CASP7; Caspase 7, CFLAR; CASP8 and FADD like apoptosis regulator, MAPK8; Mitogen-activated protein kinase 8, MAPK14; Mitogen-activated protein kinase 14, MAP2K3; Mitogen-activated protein kinase kinase 3, MAP3K5; Mitogen-activated protein kinase kinase kinase 5, TRADD; TNFRSF1A associated via death domain, RIPK1; Receptor interacting serine/threonine kinase 1, PMT1; Protein O-mannosyltransferase-1, AKT1; AKT serine/threonine kinase 1, MOAP1; Modulator of apoptosis 1, PEX5; Peroxisomal biogenesis factor 5, CASP9; Caspase 9, SIVA1; SIVA1 apoptosis inducing factor

Apoptosis-mediated cell death and mitosis ensure the maintenance of cell numbers and balanced homeostasis. Three pathways regulate apoptosis: extrinsic (death receptor), intrinsic (mitochondrial), and perforin/granzyme B [4, 108]. These pathways communicate via initiator and executor CASPs, which are a class of cysteine proteases. Two pathways are known to activate CASPs, including activation of death receptors [Fas cell surface death receptor (FAS) and TNF receptor superfamily member 1A (TNFRSF1A)]. The extrinsic and intrinsic pathways activate initiator CASPs, such as caspase 8 (CASP8) and CASP9, and executor CASPs, including CASP3 and CASP7 (Fig. 5) [109, 110]. In the extrinsic pathway, cell death signals are generated extracellularly, whereas in the intrinsic pathway, triggers are intracellular [4, 111]. The intrinsic mitochondrial pathway activates the pro-apoptotic BCL2 apoptosis regulator (BCL2) family of proteins, increases mitochondrial outer membrane permeability, and releases cytochrome C (CYCS). CYCS regulates apoptosome formation, which consists of oligomers of apoptotic peptidase-activating factor 1 (APAF1) and pro-CASP9. This leads to the activation of CASP9, followed by the subsequent activation of CASP3 and CASP7 (Fig. 5) [112–114]. In addition, a CASP-independent signaling pathway mediated by cytotoxic T cells is initiated by granzyme A (GZMA).

Mitogen-activated protein kinases (MAPKs) are essential for phosphorylation during apoptosis. During apoptosis mediated by mitogen-activated protein kinase 8 (MAPK8/JNK1) and mitogen-activated protein kinase 14 (MAPK14/p38), stress signals activate MAP kinases such as mitogen-activated protein kinase kinase kinase 1 (MAP3K1/MEKK1) and mitogen-activated protein kinase kinase kinase 5 (MAP3K5/ASK1) (Fig. 5). A cascade of kinases triggers mitogen-activated protein kinase kinase 3 (MAP2K3/MKK3), which then activates MAPK8 or MAPK14 [115, 116]. Ultimately, MAPK8 and MAPK14 activate receptor- or mitochondria-mediated apoptotic pathways by increasing the FAS ligand (FASLG), a TNF family protein, and activating or inactivating the BCL2 family of pro- and anti-apoptotic proteins (Fig. 5) [117, 118].

## TRIM proteins at the helm: influencing apoptosis via MAPKs

**Fig. 6:**
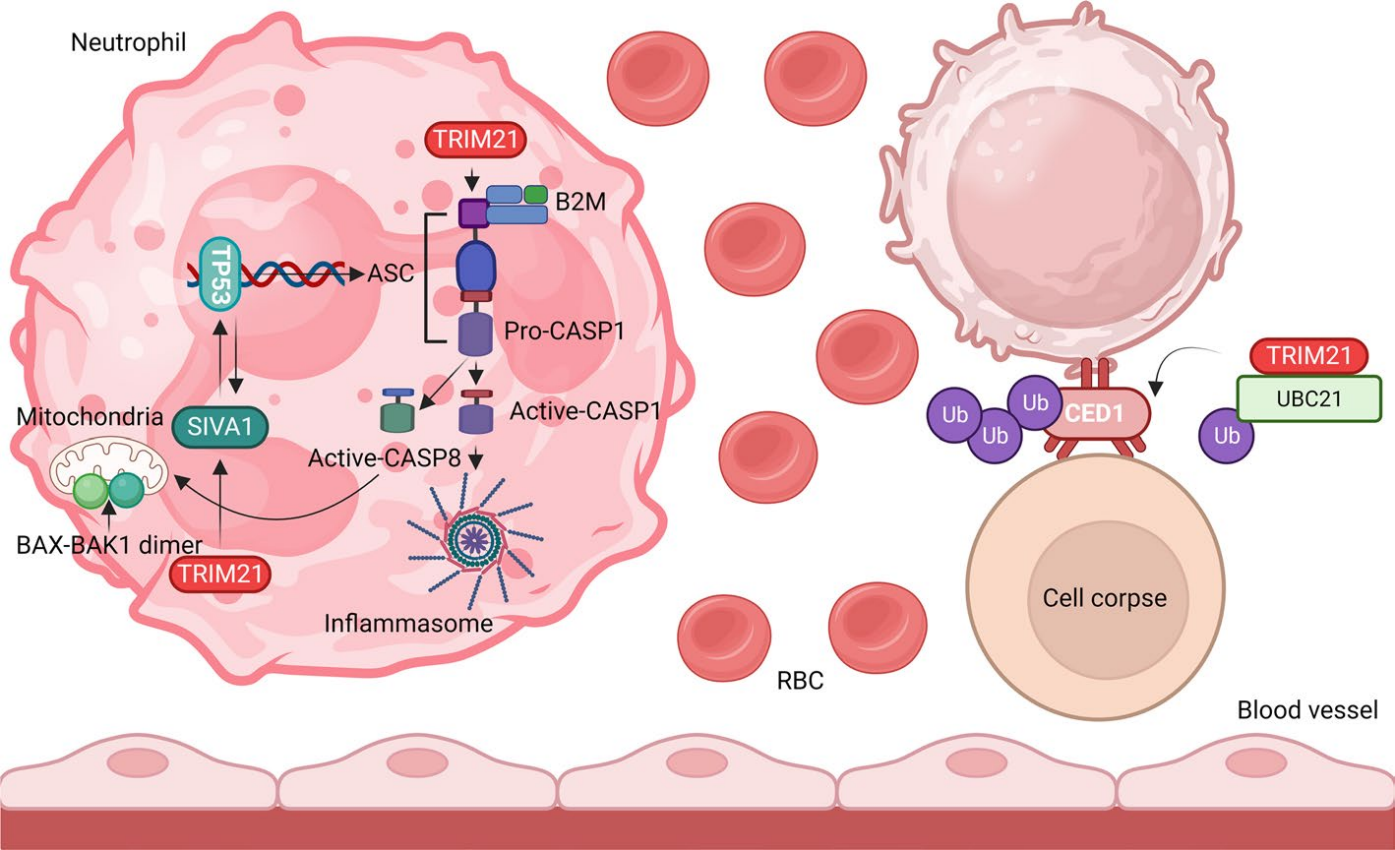
TRIM21 regulates TP53-mediated apoptosis and inflammasome activation. TRIM21 activates the formation of mature CASP8 and CASP1 and activates the inflammasome. TRIM21 also activates BAX-BAK1-mediated mitochondrial membrane pore formation and SIVA1- and TP53-mediated ASC transcription. In C. elegans, TRIM21 induces ubiquitination of CED1 to form a cell corpse. TRIM21, tripartite motif containing 21, TP53; Tumor protein p53, CASP8, Caspase 8, CASP1; Caspase 1, CASP3; Caspase 3, CASP7; Caspase 7, BAX; BCL2 associated X, apoptosis regulator, BAK1; BCL2 antagonist/killer 1, SIVA1; SIVA1 apoptosis-inducing factor, ASC/CARD; Apoptosis-associated speck-like protein containing a caspase recruitment domain, *C. elegans*; *Caenorhabditis elegans*, CED1; Cell death abnormality protein 1, B2M; Beta-2-microglobulin, TRIM21; Tripartite motif containing 21, Ub; Ubiquitin, UBC21; Ubiquitin-conjugating enzyme 21, RBC; Red blood cell

Although apoptosis is physiologically critical for processes such as inflammation, immunity, development, and remodeling, excessive apoptosis leads to many pathological conditions. New insights point to the roles of different TRIM proteins in cell death and survival under normal and diseased conditions. Oxidative stress-responsive kinases such as MAP3K5 regulate stress-induced apoptosis through mitochondria-dependent CASP activation [4, 119]. Recently, Hirata et al. (2017) discovered that TRIM48 degrades protein arginine methyltransferase 1 (PRMT1), a negative regulator of MAP3K5 (Fig. 5). PRMT1 is an enzyme that posttranslationally methylates arginine (R) in proteins. This increases the interaction between thioredoxin (TXN) and MAP3K5, thereby deactivating MAP3K5. When PRMT1 is degraded by TRIM48, its repression is released, activating MAP3K5 and consequently inducing cell death [119, 120]. Death domain-associated protein (DAXX) interacts with MAP3K5. It is a nuclear protein associated with the PML nuclear body scaffold (PML), involving TRIM19 (Fig. 5). DAXX interacts with proapoptotic receptors, such as FAS and transforming growth factor beta receptor 2 (TGFBR2), and regulates downstream protein functions (Fig. 5). It facilitates the recruitment of Fas associated via the death domain (FADD) and sequentially activates downstream CASPs, along with CASP8 (Fig. 5). These events are crucial for the MAPK8 signaling pathway [121]. In neuronal cells, the FAS-DAXX pathway activates MAPK8, whereas in nonneuronal cells, it activates MAPK14. DAXX interacts with TRIM21 in conjunction with another proapoptotic protein, caspase-8-associated protein 2 (CASP8AP2), and TRIM21 facilitates the movement of DAXX from the nucleus to the cytoplasm (Fig. 5). DAXX binds to the B30.2 domain of TRIM21, and CASP8AP2 binds to its CC domain (Fig. 5). Both interactions play an essential role in pathological conditions related to Sjögren's syndrome and systemic lupus erythematosus (SLE), in which TRIM21 is recognized as an autoantigen and is likely to promote apoptosis [122]. TRIM21 plays a crucial role in regulating anti-apoptotic regulators, specifically CASP8 and the FADD-like apoptosis regulator (CFLAR/cFLIP), as depicted in Fig. 5. It facilitates ubiquitination and subsequent degradation of CFLAR, particularly the mutant forms arising from readthrough mutations and stop codon skipping. Shibata et al. (2015) revealed that TRIM21 rapidly degrades mutant CFLAR, leading to apoptosis in hepatocytes by modulating the transcription of CFLAR isoforms through NFKB signaling. TRIM21 overexpression triggers CASP8-mediated apoptosis by disrupting CFLAR signaling (Fig. 5) [123]. Known as a universal proapoptotic protein, TRIM21 operates via a tumor protein p53 (TP53)-independent signaling pathway, targeting the anti-apoptotic protein BCL2. In individuals with systemic lupus erythematosus (SLE) and Sjogren’s syndrome, increased TRIM21 expression downregulates BCL2, promotes extensive apoptosis, and compromises intracellular immunity. Additionally, TRIM21 autoantigen overexpression is linked to increased apoptosis and reduced cell proliferation, fostering autoimmune B and T-cell responses, especially in patients with rheumatic diseases [124]. Yuan et al. elucidated a novel mechanism involving TRIM21 in regulating apoptotic cell death by maintaining the levels of cell death abnormal protein 1 (CED1). TRIM21, in coordination with UBC21, orchestrates the polyubiquitination of CED1, leading to its subsequent proteasomal degradation (Fig. 6) [125]. Previously, we discussed that TRIM21 promotes autophagy to resolve stress granules [126]. TRIM21 also functions with argonaute RISC component 4 (AGO4) to induce apoptosis in a newly discovered regulatory AGO4-TRIM21-GRP78 axis where AGO4 stabilizes TRIM21 to promote K48-linked ubiquitination of heat shock protein family A (Hsp70) member 5 (HSPA5), resulting in increased apoptosis and decreased autophagy by activating MTOR signaling [126, 127].

## Guardians of cell fate: TRIM proteins and their regulation of TP53-dependent and independent apoptosis

TRIM69 is a critical protein predominantly expressed during spermatogenesis. Germ cell homeostasis is reportedly maintained by triggering apoptosis in response to elevated TRIM69 levels. Increased TRIM69 expression induces the expression of apoptosis regulators such as BCL2-associated X, apoptosis regulator (BAX), CASP2, and receptor-interacting serine/threonine kinases (RIPKs) (Fig. 5) [128]. BAX and BCL2 antagonist/killer 1 (BAK1) are proapoptotic proteins that activate the intrinsic apoptotic pathway. The regulators of these proteins are complex and are not yet fully understood. In response to apoptotic stimuli, cytosolic BAX translocates to the mitochondria, resulting in permeabilization of the mitochondrial outer membrane (MOM) (Fig. 5). Thereafter, CYCS is released from the mitochondrial membrane, causing APAF1 to oligomerize and form an apoptosome [129]. Interestingly, TRIM69 was found in the nucleus and colocalized with TRIM19. The colocalization of TRIM69 with TRIM19 in the nucleus may imply a potential functional relationship or regulatory interplay between these two proteins in nuclear-related processes. The signal associated with MAPK14 phosphorylation-dependent nuclear localization is responsible for its localization and PML-mediated apoptosis. Although the role of CASP2 in apoptosis is not well defined, its similarity to that of CASP9, the initiator of the intrinsic signaling pathway, indicates its involvement in apoptosis [130]. Furthermore, TRIM16 induces apoptosis by directly binding to CASP2 and modulating its activity. The caspase recruitment domain family member (CARD) domain in RIPKs may be responsible for the activation of CASP2, suggesting a noncanonical role of CASP2 in TRIM69- and TRIM19-mediated apoptosis (Fig. 5) [131].

In contrast, TRIM69 overexpression did not increase apoptosis in zebrafish. However, loss of TRIM69 in zebrafish induces TP53-dependent apoptosis. TP53 is a tumor suppressor protein that forms an extensive signaling network in response to various cellular stressors. Most reactions result in nuclear accumulation of TP53, which regulates cell proliferation and cell death through apoptosis. TP53-mediated apoptosis occurs through both extrinsic and intrinsic pathways. Loss of TRIM69 follows an intrinsic cell death pathway initiated by TP53, and TRIM69 expression rescues ongoing apoptosis (Fig. 5) [132].

New findings have confirmed that the activating signaling cointegrator (ASC) is a regulator of TP53 targets. ASC comprises a pyrin domain and a CARD. In addition to being an important inflammatory molecule, it regulates apoptosis. It interacts with TRIM20, an inflammation-regulating cytosolic protein that induces familial FMF [133]. ASC is significantly upregulated in FMF and plays an important role in neutrophil apoptosis, which is impaired in patients with FMF. TRIM20 suppresses neutrophil inflammation and apoptosis in ASCs. TRIM20 was also associated with another proapoptotic TP53-selective target gene, SIVA1 [134] (Fig. 5). TRIM20 orchestrates the interaction between SIVA1 and ASC spots while modulating its function. All of these functions have been suggested to have pathophysiological consequences in FMF. In addition, it also indicates the role of neutrophils and monocytes in which SIVA1, pyrin, and ASC are coexpressed [135]. However, FMF-induced *TRIM20* mutations did not affect SIVA1-mediated apoptosis. Another study found that SIVA1 was not associated with TRIM20 in an FMF gene network constructed using a gene signature curated in the literature. Although many reports have shown increased, widespread neutrophil cell death in FMF with mutant TRIM20, no single and discrete apoptotic pathways have been elucidated. However, TRIM20-mediated apoptosis protects cells from severe inflammation in acute cases [136].

There are several BAX activity enhancers, such as modulator of apoptosis 1 (MOAP1). MOAP1 increases BAX activity when damage is induced and is degraded by a multisubunit E3 ligase called the anaphase-promoting complex (ANAPC). MOAP1 has a short half-life, during which it stimulates the mitochondria to release CYCS through BAX activation (Fig. 5). TRIM39 can stabilize MOAP1 and increase its levels in mitochondria by inhibiting its polyubiquitination and degradation, thus regulating apoptosis. Additionally, when cells are exposed to etoposide, a chemotherapeutic drug, TRIM39 can detect etoposide-induced DNA damage and promote cell death. Cells overexpressing TRIM39 are less affected by etoposide because of the molecular control of MOAP1 activity [137–139]. In addition, it has also been reported that TRIM22 plays a crucial role in monocyte apoptosis in sepsis models. Initially, an increase in apoptosis was not observed in LPS-primed TRIM22-overexpressing monocytes. After sensitization to the apoptotic inducer staurosporine, significant oligomerization of the pro-apoptotic protein BAK1 was observed along with an increase in CASP9 and CASP3 levels [28]. In contrast to BAX, which is mainly found in the cytosol, BAK1 is a membrane protein that is normally localized in the MOM. BAK1 then migrates to mitochondria during apoptosis and activates the intrinsic apoptotic pathway [140, 141]. The correlation between TRIM22 reduction and a decrease in BAK1 levels suggests decreased apoptosis and immunosuppression in sepsis (Fig. 5) [28].

TRIM27 has been reported to play a role in apoptotic signaling pathways involving MAPKs and CASPs, which do not undergo mitochondrial events. Overexpression of TRIM27 leads to extensive cell death, which is blocked by a CASP8 inhibitor. Although the RBCC domain of TRIM27 was shown to trigger MAPK8, MAPK14, and CASPs, it did not induce the release of CYCS from the mitochondria (Fig. 5) [142]. Furthermore, TRIM27 positively regulated TNF-induced apoptosis. TNF is a pleiotropic cytokine that induces cell death in tumor cells. TNF-induced apoptosis activates CASP8 via two distinct pathways involving baculoviral IAP repeats 2/3 (BIRC2/3) and CFLAR [143]. Although TRIM27 is a prominent Ub ligase, cytosolic TRIM27 is known to deubiquitinate RIPK1. Further studies have shown that TRIM27 forms a complex with ubiquitin-specific peptidase 7 (USP7) in the cytosol. The cycle of ubiquitination of USP7 by TRIM27 and deubiquitination of RIPK1 by the TRIM27-USP7 complex are the basis for TRIM27-regulated TNF-mediated apoptosis. Deubiquitination of RIPK1 stabilizes this protein, leading to its activation. Activation of RIPK1 associates it with FADD and pro-CASP8 [144]. Pro-CASP8 forms active CASP8, which cleaves Pro-CASP3 into its active effector, CASP3, to induce apoptosis (Fig. 5). Li et al. (2021) reported that TRIM27 can interact with TP53 to suppress apoptosis and inflammation in cardiac ischemia and perfusion injury [145]. TRIM34 localizes to the mitochondria and can cause depolarization of mitochondrial membrane potential, release CYCS, and induce apoptosis [146].

In addition to TRIM27, TRIM32 is known to prime cells for TNF-induced apoptosis. TRIM32 ubiquitinates X-linked inhibitor of apoptosis (XIAP), an apoptotic suppressor that inhibits CASP3 and CASP7 (Fig. 5). Furthermore, XIAP exerts its anti-apoptotic function by binding to the TNFRSF1A-associated factors TRAF1 and TRAF2 [27]. Thus, TRIM32 regulates XIAP levels by mediating proteasomal degradation and promoting apoptosis. In another study, its suppression was shown to benefit traumatic brain injury in mouse models by enhancing neuronal recovery via tumor protein 73 (TP73)-mediated antiapoptotic pathways [147]. The TRIM32 homolog thin (TN) in Drosophila is involved in larval development. TRIM32 also induces muscle cell death by controlling apoptosis. It ubiquitinates and degrades antiapoptotic factors such as diaphanous-related formin 1 (DIAPH1), Drosophila Nedd2-like caspase/Apoptotic peptidase activating factor 1 (DRONC/APAF1), and death-associated APAF1-related killer (DARK/CYCS) and eventually promotes apoptosis through CASP regulation (Fig. 5) [148].

Several TRIM proteins and apoptotic factors are localized in the nucleus, with TRIM19 forming PML nuclear bodies. These nuclear bodies are significant contributors to fundamental cellular processes such as tumor suppression, apoptosis regulation, DNA repair, and transcriptional control (Fig. 5) [149, 150]. TRIM19 is associated with the ER and mitochondria-associated membranes (MAMs) in the cytosol and regulates calcium signaling [151]. It acts as a mediator of the crosstalk between the ER and mitochondria in MAMs, where it monitors calcium efflux from the ER to the mitochondria and, in turn, controls Ca2 + -dependent apoptosis [152]. Both nuclear and cytosolic localization of TRIM19 allow it to act as a platform for protein recruitment, assembly of their posttranslational modifications, and regulation of the availability and function of other apoptotic factors (Fig. 5) [153]. Thus, TRIM19 is a versatile, pleiotropic regulator of apoptosis. TRIM19 is primarily involved in TP53-mediated apoptosis modulated by DAXX. TP53 and DAXX interact directly with TRIM19. Furthermore, similar to TP53, TRIM19 functions as a transcriptional coactivator of many apoptotic genes. It works in concert with TP53 to induce various factors, such as BAX and p21 (RAC1)-activated kinase 2 (PAK2). The absence of TRIM19 disrupts apoptosis-related TP53 function, suggesting its critical role in TP53-mediated cell death [154]. DAXX and TRIM19 coordinate under cellular stress, with DAXX localizing to PML nuclear bodies and performing its functions only when TRIM19 is activated [155]. In addition to DAXX, there are other posttranslational modulators, such as small ubiquitin-like modifier 1 (SUMO1), which aid in SUMOylation of TRIM19 and formation of PML nuclear bodies (Fig. 5). SUMO1 levels and functions are directly proportional to DAXX levels in PML nuclei, which are transiently trapped in response to SUMOylated TRIM19. The greater the SUMOylation, the greater the DAXX flux, and the lower the FAS-mediated apoptosis [156]. Meinecke et al. showed that synovial fibroblasts in rheumatoid arthritis become resistant to cell death induced by FASLG. This is due to increased SUMO1 levels and a consequent decrease in nuclear SUMO-specific peptidase 1 (SENP1) levels. SUMO1 traps DAXX in PML bodies, preventing it from exerting proapoptotic effects. Furthermore, a reduction in SENP1 levels leads to reduced proteasomal degradation of SUMO1 and thus increased function. TRIM19 SUMOylation is central to its ability to recruit factors to PML nuclei and modulate apoptosis [156]. Hayakawa et al. (2004) took a contrasting view that phosphorylation and concomitant SUMOylation of TRIM19 induce and enhance apoptosis in arsenic trioxide-treated cells [157]. The differences in apoptotic behavior in response to similar changes can be determined by context and specific cases, as they vary widely in different diseases [158].

TRIM19 also promotes apoptosis in a TP53-independent manner, as has been shown in multiple studies. Both TP53-dependent and TP53-independent apoptosis have been reported under gamma irradiation stress, involving DNA damage checkpoint kinase 2 (CHK2). The TP53-independent signaling pathway is dependent on ATM serine/threonine kinase (ATM) [159]. Here, TRIM19/TNF-induced cell death was characterized by events such as DNA fragmentation and activation of CASP3, CASP7, and CASP8. Thus, TRIM19 acts as an NFKB transcriptional repressor mediated by the RELA protooncogene, the NF-kB subunit (RELA), and regulates apoptosis by repressing the NFKB signaling pathway (Fig. 5) [160].

In acute promyelocytic leukemia (APL), TRIM19 fuses with the retinoic acid receptor alpha gene (RARA) to form the PML-RARA gene. Consequently, this fusion gene is cleaved by elastase-neutrophil-expressed protein (ELANE), resulting in cytosolic TRIM19 without a nuclear localization signal. TRIM19 lacking a nuclear localization signal enhances the transcription of BCL2 and MYC proto-oncogenes and the bHLH transcription factor (MYC) and downregulates BAX, thereby inhibiting leukemia cell apoptosis [161]. The absence of TRIM19 makes cells resistant to death. These features can be used to combat various inflammatory and apoptotic pathologies [162].

The BCL2 family contains various pro-apoptotic and anti-apoptotic proteins that coordinate cell fate [113]. The ubiquitination and proteasomal degradation of antiapoptotic factors are efficient pathways for regulating apoptosis [163]. Neuronal regulation of apoptosis is mediated by MCL1, a member of the BCL2 protein family. Phosphorylated MCL1 is targeted by the Ub ligase TRIM17 in neurons. Cellular TRIM17 levels affect phosphorylation- and ubiquitination-mediated degradation of MCL1. TRIM17 expression activates the intrinsic pathway that leads to neuronal apoptosis, which is attenuated when either BAX is absent or MCL1 is unphosphorylated [164]. Similar to MCL1, BCL2-related protein A1 (BCL2A1) is an antiapoptotic member of the BCL2 family. TRIM17 stabilizes BCL2A1 levels in contrast to its depletion of MCL1. TRIM28 acts as a Ub ligase for BCL2A1. TRIM17 releases BCL2A1 by preventing ubiquitination of BCL2A1 by TRIM28. These regulatory phenomena have important consequences in melanoma treatment, where either *TRIM28* overexpression or *TRIM17* knockout lowers BCL2A1 protein levels and restores melanoma cell susceptibility to B-Raf protooncogene and serine/threonine kinase (BRAF)-directed therapy [165].

Neuronal apoptosis is also regulated by the availability of apoptotic factors such as nuclear factor of activated T cells (NFAT) transcription factors, which are transcriptionally controlled. Nuclear factor of activated T cells 3 (NFATC3) and NFATC4 are two important transcription factors whose levels are regulated by TRIM17 by channeling their transport between the nucleus and cytoplasm via a calcium-dependent pathway (Fig. 5). Although TRIM17 inhibits NFATC3 and NFATC4, both mechanisms have opposite effects. While inhibition of NFATC3 alleviates apoptosis, inhibition of NFATC4 exacerbates it. A feedback loop involving NFATC3 promotes TRIM17 transcription by binding to its promoter, which results in increased apoptosis [166]. Another group of nuclear factors, nuclear receptor subfamily 4, group A, member 1 (NR4A1), a member of the NR4A subfamily of nuclear receptors, also regulates apoptosis via TRIM13, which acts as a Ub ligase. Although TRIM13 is not the sole control switch for NR4A1, it plays a crucial role in setting NR4A1 levels in conjunction with casein kinase 2 alpha 2 (CSNK2A2). In addition, it also controls the levels of the MDM2 protooncogene (MDM2) and AKT serine/threonine kinase 1 (AKT1), thereby increasing the levels of TP53, BAX, and cyclin-dependent kinase inhibitor 1A (CDKN1A), leading to increased apoptosis under ionizing radiation [167, 168]. Diao et al. reported that TRIM14 is highly expressed in human cervical cancer cells and regulates cell proliferation and death through the AKT1 pathway [169]. In contrast, TRIM14 is inhibited to protect against cerebral ischemia injury, which is regulated by NFKB/NLRP pathway-mediated apoptosis [170]. Another study reported that TRIM8 depletion alleviated the oxidative stress caused by hypoxia/reoxygenation. It functions by triggering the phosphatidylinositol-4,5-bisphosphate-3-kinase catalytic subunit alpha (PIK3CA)/AKT1 signaling pathway and inhibits apoptosis and pyroptosis. TRIM8 also regulate NFKB and JAK-STAT pathways and can acts as a oncogene or tumor suppressor [171, 172].

TRIM37 is associated with peroxisomal function and, together with peroxisomal biogenesis factor 5 (PEX5), regulates peroxisomal protein transport. It monoubiquitinates and stabilizes PEX5 to perform its functions. TRIM37 mutations impair peroxisomal function via PEX5 and promote CASP-dependent apoptosis [173]. Phenotypically and pathologically, these manifest as diseases, such as mulberry nanism, which is now grouped as a peroxisome biogenesis disorder (Fig. 5).

## Role of TRIMs in necroptosis and necrosis regulation

Necroptosis is a cell death process that promotes inflammation. Receptor interacting serine/threonine kinase 1 (RIPK1) kinase activity is critical for regulating necroptosis and cell-autonomous cytokine production. A recent study used mass spectrometry to analyze phosphorylation events during necroptosis and identified a RIPK1-dependent phosphorylation pattern associated with proinflammatory cytokine production, specifically marked by p-S473 TRIM28. Activation of mitogen-activated protein kinase 14 (MAPK14), mediated by oligomerized mixed lineage kinase domain like pseudokinase (MLKL), was found to promote the phosphorylation of S473 TRIM28, which in turn mediates inflammation during late necroptosis. This study provides insight into the mechanisms by which RIPK1 kinase activation controls the inflammatory response during necroptosis [174]. Necroptosis increases tumor immunogenicity and can be targeted for cancer immunotherapy. This leads to the synthesis of inflammatory proteins, which facilitate antitumor immune responses. TRIM28 has been identified as a corepressor that regulates transcriptional activity during necroptosis. Activated RIPK3 phosphorylates TRIM28, inhibiting its chromatin-binding activity and contributing to the transactivation of NFKB and other transcription factors. The derepression of TRIM28 in cancer cells leads to increased immunostimulatory cytokine production in the tumor microenvironment, which contributes to robust cytotoxic antitumor immunity [175]. Reactive oxygen species (ROS) are one of the main causes of cardiac injury following myocardial infarction. The clinical application of antioxidants has shown limited effects in protecting the heart against ischemia–reperfusion (IR) injury. Tripartite motif containing 72 (TRIM72) is a muscle-specific protein belonging to the tripartite motif containing family of proteins. The role of TRIM72 in the regulation of necroptosis following IR injury in the heart was investigated. TRIM72 mediates cardioprotection by inhibiting necroptosis [176].

Eugenio et al. used a combination of TNF-related apoptosis-inducing ligand (TRAIL), Z-VAD-FMK (pan-caspase inhibitor), and birinapant (SMAC mimetic) to characterize the necroptotic death signal induced by TRAIL. They identified TRIM21 as a new partner in the TRAIL-induced necrosome protein complex. TRAIL is a member of the TNF superfamily that can induce apoptosis and necroptosis in cancer cells. TRAIL selectively induces apoptosis in cancer cells, while sparing normal cells, making it a potential tool for cancer therapy. However, some cancer cells are resistant to TRAIL-induced cell death because of the high expression of anti-apoptotic factors. Second mitochondria-derived activator of caspase (SMAC) mimetics have been developed to sensitize resistant cancer cells to apoptosis by inhibiting inhibitors of apoptosis proteins (IAPs). The combination of TRAIL with SMAC mimetics and a pan-caspase inhibitor can activate necroptosis. TRIM21 plays a role in TRAIL-induced necroptosis. Decreased or abolished TRIM21 expression conferred resistance to TRAIL-induced necroptosis, whereas TRIM21 overexpression sensitized cells to TRAIL-induced death [177]. Some TRIMs are important in necrosis but not in apoptosis. One such example has been observed in injured heart muscle cells. Necrosis and apoptosis were examined in response to H_2_O_2_ and staurosporine treatments. Necrosis resulted in an increase in the number of several novel markers of necrosis, including TRIM72, heat shock protein 90 alpha family class A member 1 (HSP90AA1), and actin alpha 1 (ACTN1), suggesting that TRIM72 plays a role in the necrotic pathway but not in apoptosis. It also includes many classic necrotic markers, such as lactate dehydrogenase A (LDHA), high-mobility group box 1 (HMGB1), myoglobin (MB), enolase 1 (ENO1), and YWHA proteins [178]. These diverse results suggest that TRIMs have diverse functions that are dependent on mediators, cellular requirements, and many other intermediate factors that ultimately determine cell fate.

## Translating the diverse functions of TRIM proteins into clinical significance

### Cancer therapies

Understanding the role of TRIM proteins in regulating cell death and survival could provide potential targets for cancer therapy. It is well established that many important molecular biomarkers of cancer, such as BRCA1 DNA repair associated (BRCA1), BRCA2, TP53, phosphatase and tensin homolog (PTEN), and erb-b2 receptor tyrosine kinase 2 (ERBB2), are linked to autophagy and apoptosis [179]. However, specific TRIMs interact with these proteins to regulate their functions. For example, BRCA1 negatively regulates autophagic vacuole formation in MCF-7 breast cancer cells, and downregulation of *BRCA1* promotes breast cancer growth via the upregulation of autophagy [180]. Low TRIM21 expression is correlated with poor overall survival in triple-negative breast cancer patients, and knockout of *TRIM21* promotes the proliferation, migration, and invasion capability of triple negative breast cancer (TNBC) cells [181]. Recently, Huang et al. demonstrated that the combination of olaparib and sorafenib results in DNA damage, cell cycle arrest, and apoptosis in TNBC cells. BRCA1 has been identified as a ubiquitination substrate for TRIM21, and the upregulation of BRCA1 after Olaparib treatment may explain the relative resistance of BRCA1-proficient TNBC cells to olaparib. Sorafenib's effectiveness in combination therapy may be attributed to the TRIM21 mediated degradation of BRCA1 [182, 183]. Studies have shown that TP53 is involved in apoptosis, autophagy, and cancer, and its regulatory role is mediated by TRIM proteins such as TRIM11 and TRIM67. TRIM11 downregulates TP53 in hepatocellular carcinoma (HCC), making it a potential therapeutic target [184]. Conversely, *TRIM67* downregulation in colorectal cancer is associated with poor survival, and its knockout in ApcMin mice increases the incidence and severity of colorectal tumors. The knockout of *TRIM67* in mice results in the acceleration of colorectal cancer development, indicating its potential as a therapeutic target for this type of cancer [185]. PTEN modulates the PI3K/AKT1/MTOR pathway, affecting both the cellular processes. TRIM27 promotes proliferation, inhibits apoptosis, and enhances glucose uptake in esophageal squamous cell carcinoma (ESCC) cells. TRIM27 interacts with PTEN, leading to its ubiquitination and degradation, and is involved in the PI3K-AKT1 signaling pathway [186]. Accumulating evidence suggests that manipulating TRIM proteins to modulate apoptosis and autophagy may offer new approaches for inducing cancer cell death or preventing uncontrolled growth.

### Neurodegenerative disorders

Diseases such as Alzheimer's, Parkinson's, and Huntington’s are characterized by dysfunctional autophagy and apoptosis, and TRIM proteins play a crucial role in these processes. Targeting TRIM proteins could potentially lead to new therapies to regulate cell death and remove the toxic protein aggregates seen in these disorders. For example, Huang et al. demonstrated that silencing *TRIM10* decreased cell apoptosis and ROS levels in a cellular model of Parkinson's disease (PD), suggesting a potential role for TRIM10 in PD treatment. TRIM10 promotes the degradation of dual specificity phosphatase 6 (DUSP6) by increasing its ubiquitination, leading to inhibition of DUSP6 expression. This study provides new insights into the role of TRIM10 in PD and suggests potential approaches for future clinical trials, such as investigation of TRIM10 and DUSP6 inhibitors. Several studies have also shown that TRIM16 heterodimerizes with other TRIM family members and plays a role in autophagy and endomembrane damage homeostasis [69, 187]. TRIM16 plays a crucial role in facilitating the clearance of protein aggregates by interacting with galectin-3 (LGALS3) and orchestrating autophagy. It contributes to the clearance of protein aggregates by interacting with other TRIM proteins and galectins, which are involved in the cellular response to misfolded proteins. This process aids the degradation of misfolded proteins and prevents their accumulation [67, 69, 84]. TRIM16 has also been implicated in the regulation of immune activation and autophagy, making it a potential therapeutic target for tauopathies, which are characterized by intracellular neurofibrillary tangles composed of hyperphosphorylated filamentous tau proteins [67]. Mutations in the tau-encoding microtubule associated protein tau (MAPT) gene can cause heritable tauopathies. TRIM11 was found to retain tau in its functional soluble state, which differs from other protein quality control factors. TRIM11 levels are significantly reduced in the brains affected by sporadic Alzheimer's disease, implying its possible involvement in the pathogenesis of the disease. Using intracranial adeno-associated viral delivery, TRIM11 provided strong protection against tau-related pathology, cognitive decline, and motor impairments in multiple tauopathy mouse models [188].

### Infections and immune system modulation

Viruses often interfere with cellular mechanisms, including autophagy and apoptosis, to ensure their survival and replication. TRIM5 has been reported to interact with cellular regulators of autophagy and deliver restriction-sensitive retroviral capsids to autophagosomes for destruction. However, the exact mechanism through which autophagy contributes to TRIM5-mediated retroviral restriction remains unclear [189]. To address this, Imam et al. demonstrated that autophagy is not necessary for TRIM5α to restrict retroviruses but is essential for the degradation of TRIM5α. These findings suggest that the effector functions of TRIM5α can be distinguished from its degradation, which has implications for understanding the mechanisms of other TRIM family members. These results provide new insights into the regulation of TRIM5α and may lead to the development of novel antiviral strategies targeting retroviral infections [190]. A different study discovered that next-generation sequencing showed that TRIM25 is upregulated during rabies virus and HEP-Flury infections. Decreasing TRIM25 levels enhances HEP-Flury production, but if it increases, it suppresses HEP-Flury replication. Additionally, knockdown of *interferon alpha 1 (IFNA1)* and *interferon beta 1 (IFNB1)* weakened the antiviral response induced by TRIM25 overexpression and increased rabies virus (RABV) production [191]. Investigating TRIM proteins involved in these pathways may provide insights into controlling viral infections by targeting specific host factors. TRIMs influence immune response regulation and viral evasion by modulating the signaling pathways of antiviral immune responses, such as type I IFN, NFKB, JAK/STAT, and zinc and ring finger 2 (ZNRF2). However, viruses can counteract TRIM activity or utilize TRIMs for their replication, leading to immune evasion. Understanding the mechanisms by which TRIMs participate in viral immune evasion can help to identify new molecular targets for treating and preventing viral infectious diseases. It is crucial to study the dynamic interactions between TRIMs and viral proteins to develop strategies to combat viral infections [192]. Romagnoli et al. found that TRIM32, a novel player involved in the intracellular response to *Mycobacterium tuberculosis* (*M. tb*) infection in macrophages, promotes autophagy-mediated *M. tb* degradation. This suggests that TRIM32 is a potential target for host-directed tuberculosis therapies. TRIM32 plays a role in regulating cellular processes beyond muscle cells and in the innate immune response to viral infections. In addition, it has been identified as an important regulator of the cyclic GMP-AMP synthase-stimulator of interferon response cGAMP interactor 1 (CGAS-STING1) pathway for cytosolic DNA sensing. The expression or activity of TRIM32 may serve as a biomarker for stratifying tuberculosis stages or as a target for host-directed therapies. Notably, TRIM32 expression has been reported to decrease leukocytes from tuberculosis patients with tuberculosis compared to healthy controls and subjects with tuberculosis infection. These findings suggest that targeting TRIM32 could be a promising strategy for enhancing the host immune response to *M. tb* infection and developing new therapeutic approaches for tuberculosis [193].

### Novel therapeutic targets and precision therapies

Understanding the roles of TRIM proteins and their specific isoforms in autophagy and apoptosis could lead to the creation of a personalized set of biomarkers that play a critical role in therapeutic interventions. Therefore, TRIM proteins may serve as potential drug targets. The development of drugs that regulate the functions of these proteins could offer new ways to control autophagy and apoptosis, leading to therapeutic interventions for various diseases. Identifying patients with dysfunctional TRIM proteins and clarifying their roles are crucial for customizing therapies that restore or modulate these pathways, potentially improving patient outcomes. Identifying the specific TRIM proteins involved in these processes and their mechanisms of action in autophagy and apoptosis are essential for further clinical translation. By identifying their precise roles and developing methods to modulate their functions, there may be opportunities for more targeted therapies for a range of diseases, potentially enhancing treatment effectiveness, and improving patient outcomes.

## Conclusions and perspectives

The complex and varied functions of TRIM proteins in cell physiology, innate immunity, and cell death necessitate a comprehensive understanding of their mechanistic actions. This review provides a holistic view of the pivotal role of TRIM proteins in the regulation of autophagy and apoptosis, shedding light on their significance in various cellular processes. The profound implications of TRIM proteins in autophagy, apoptosis, and innate immunity open exciting prospects for future biomedical research and therapeutic developments.

TRIM proteins have emerged as vital posttranslational modifiers regulating a broad spectrum of critical biological processes and immune responses. These proteins play essential roles in modulating viral and bacterial immunity, cell stress responses, proliferation, autophagy, apoptosis, differentiation, transcription, and DNA repair. The increase in TRIM protein numbers in evolutionarily complex multicellular organisms, compared to less complex ones, highlights their pivotal involvement in regulating diverse processes beyond cell proliferation and differentiation.

Recently, the multifaceted roles of TRIM proteins in autophagy and apoptosis have come to light. The presence of the LIR motif within the CC domain of specific TRIM proteins suggests their function as scaffolding proteins, facilitating interactions with autophagy receptors to efficiently target substrates for delivery to autophagosomes. These receptors interact with the ATG8 family of proteins, which are essential for phagophore and autophagosome formation. The presence of the LIR motif in TRIM proteins suggests that many TRIM proteins may also serve as autophagy receptors, proficient at recognizing and efficiently directing substrates to autophagosomes for degradation. Their involvement in Ub-mediated targeting of bacteria to the UPS underscores the critical role of TRIMs in regulating UPS-mediated bacterial and protein aggregate targeting.

Based on this evidence, it is reasonable to hypothesize that additional TRIM proteins may function as autophagy receptors, aiding the Ub-mediated targeting of various substrates to autophagosomes for lysosome-mediated degradation. These substrates include bacteria, viruses, dysfunctional organelles, misfolded proteins and immunoregulators. These findings indicate that TRIM proteins play both positive and negative roles in regulating innate immune signaling, such as the type I IFN response to viruses, proinflammatory responses, NFKB signaling, and antifungal responses, corroborating this assumption.

The polyubiquitination-mediated regulation of specific immune sensors also suggests that TRIMs may be involved in the Ub-mediated regulation of NFKB signaling, as previous findings have shown that many Ub regulators are involved in controlling NFKB activation and proinflammatory cytokine production. Surprisingly, many TRIM proteins were ubiquitously expressed. The widespread presence of TRIM proteins in tissues along with their capability to carry out functions through distinct domains and isoforms introduces a level of regulatory intricacy that deserves more exploration. TRIM proteins hold great potential as new therapeutic targets to combat various diseases, owing to their diverse roles in critical biological processes. Researchers have explored their molecular complexities, roles in autophagy, apoptosis, and important signaling pathways that can be manipulated for therapeutic purposes.

The significance of TRIM proteins in autophagy, apoptosis, and innate immunity has potential for biomedical research and the development of treatments. Understanding the regulation of TRIM proteins may lead to new therapeutic approaches for diseases characterized by disrupted autophagy and apoptosis, including cancer, neurodegenerative disorders, and viral infections. By manipulating TRIM proteins, we could open opportunities for targeted therapies for numerous individuals affected by these conditions.

## Data Availability

All data have been included in the manuscript.

## Abbreviations

ACTN1: Actinin alpha 1
APL: Acute promyelocytic leukemia
AGO4: Argonaute RISC component 4
ASC: Activating signal cointegrator
APL: Acute promyelocytic leukemia
ARF: ADP ribosylation factor
AKT1: AKT serine/threonine kinase 1
ANAPC: Anaphase-promoting complex
APAF1: Apoptotic peptidase activating factor 1
R: Arginine
ATM: ATM serine/threonine kinase
AMBRA1: Autophagy and beclin 1 regulator 1
ATG16L1: Autophagy related 16 like 1
ATG7: Autophagy-related 7
ATG: Autophagy-related
BIRC2/3: Baculoviral IAP repeat containing 2/3
BAK1: BAX and BCL2 antagonist/killer 1
BCL2: BCL2 apoptosis regulator
BAX: BCL2 associated X, apoptosis regulator
BCL2A1: BCL2 related protein A1
BECN1: Beclin 1
BRAF: B-Raf proto-oncogene, serine/threonine kinase
CALCOCO2: Calcium-binding and coiled-coil domain 2
CTDs: Carboxy-terminal domains
CSNK2A2: Casein kinase 2 alpha 2
CFLAR/cFLIP: CASP8 and FADD-like apoptosis regulator
CASP8: Caspase 8
CARD: Caspase recruitment domain family member
CED1: Cell death abnormality protein 1
PIK3CA: Phosphatidylinositol-4,5-bisphosphate 3-kinase catalytic subunit alpha
CHK2: Checkpoint kinase 2
CHRN: Cholinergic
CKDs: Chronic kidney diseases
CCs: Coiled coils
CGAS-STING1: Cyclic GMP-AMP synthase-stimulator of interferon response cGAMP interactor 1
CDKN1A: Cyclin dependent kinase inhibitor 1A
CYCS: Cytochrome C
COPD: Chronic obstructive pulmonary disease
DAXX: Death domain associated
DARK: Death-associated APAF1-related killer
DIAPH1: Diaphanous-related formin 1
ds: Double-stranded
DRONC: Drosophila Nedd2-like caspase
ELANE: Elastase neutrophil-expressed
EMCV: Encephalomyocarditis virus
ER: Endoplasmic reticulum
ENO1: Enolase 1
EV71: Enterovirus 71
EIF4EBP1: Eukaryotic translation initiation factor 4E binding protein 1
FMF: Familial Mediterranean fever
FADD: Fas associated via the death domain
FAS: Fas cell surface death receptor
FASLG: FAS ligands
GABARAP: GABA type A receptor-associated protein
LGALS3: Galectin 3
LGALSs: Galectins
GZMA: Granzyme A
G3BP1: G3BP stress granule assembly factor 1
HSP90AA1: Heat shock protein 90 alpha family class A member 1
HSV-1: Herpes simplex virus 1
HMGB1: High mobility group box 1
HDAC6: Histone deacetylase 6
HIV: Human immunodeficiency virus
HSPA5: Heat shock protein family A (Hsp70) member 5
IRF3: IFN regulatory factor 3
IAV: Influenza A virus
IKBKB: Inhibitor of nuclear factor kappa B kinase subunit beta
IFNA1: Interferon alpha 1
IRF8: Interferon regulatory factor 8
IFN: Interferon
IL1B: Interleukin 1 beta
IL18: Interleukin 18
KEAP1: Kelch-like ECH-associated protein 1
LDHA: Lactate dehydrogenase A
LIR: LC3-interacting region
LPS: Lipopolysaccharide
MAGEA3: MAGE family member A3
MAP1LC3: Microtubule-associated protein 1 light chain 3
MCL1: MCL1 apoptosis regulator, BCL2 family member
MDM2: MDM2 proto-oncogene
MTOR: Mechanistic target of rapamycin kinase
MAP1LC3: Microtubule-associated protein 1 light chain 3
MAMs: Mitochondria-associated membranes
MAP: Mitogen activated protein
MAPK14/p38: Mitogen-activated protein kinase 14
MAPK8/JNK1: Mitogen-activated protein kinase 8
MAP2K3/MKK3: Mitogen-activated protein kinase kinase 3
MAP3K1/MEKK1: Mitogen-activated protein kinase kinase kinase 1
MAP3K5/ASK1: Mitogen-activated protein kinase kinase kinase 5
MOAP1: Modulator of apoptosis 1
Mulibrey: Muscle-liver-brain-eye
MYC: MYC proto-oncogene, bHLH transcription factor
M. tb: Mycobacterium tuberculosis
MB: Myoglobin
NBR1: NBR1 autophagy cargo receptor
NLRP3: NLR family pyrin domain containing 3
NLRs: NOD-like receptors
NFKB: Nuclear factor KB
NFATC3: Nuclear factor of activated T cells 3
NFAT: Nuclear factor of activated T cells
NR4A1: Nuclear receptor subfamily 4 group A member 1
OPTN: Optineurin
PAK2: P21 (RAC1)-activated kinase 2
PEX5: Peroxisomal biogenesis factor 5
PIK3C3: Phosphatidylinositol 3-kinase catalytic subunit type 3
PIK3R4: Phosphoinositide-3-kinase regulatory subunit 4
Pro-CASP1: Pro-caspase-1
PCD: Programmed cell death
Pro3C: Protease 3C
PRKAA2/AMPK: Protein kinase AMP-activated catalytic subunit alpha 2
PRR: Pattern recognition receptor
RING: Really interesting new gene
RIPKs: Receptor interacting serine/threonine kinases
RARA: Retinoic acid receptor alpha
RPS6KB1: Ribosomal protein S6 kinase B1
RLRs: RIGI like receptors
S. typhimurium: Salmonella typhimurium
SQSTM1/p62: Sequestome 1
SH3GLB1: SH3 domain containing GRB2 like, endophilin B1
SUMO1: Small ubiquitin-like modifier 1
SENP1: SUMO-specific peptidase 1
SLE: Systemic lupus erythematosus
TBK1: TANK-binding kinase 1
TAX1BP1: Tax1 binding protein 1
TN: Thin
TXN: Thioredoxin
TNFRSF1A: TNF receptor superfamily member 1A
CASP8: Caspase 8
TLRs: Toll-like receptors
TFEB: Transcription factor EB
TGFBR2: Transforming growth factor beta receptor 2
TRIM: Tripartite motif containing
TSC1: TSC complex subunits 1
TRAF6: Tumor necrosis factor receptor-associated factor 6
TP53: Tumor protein p53
TP73: Tumor protein p73
Ub: Ubiquitin
USP7: Ubiquitin-specific peptidase 7
UPS: Ubiquitin‒proteasome system
ULK1: Unc-51 like autophagy activating kinase 1
XIAP: X-linked inhibitor of apoptosis
ZNRF2: Zinc and ring finger 2
